# Cochlear implant electrode design for safe and effective treatment

**DOI:** 10.3389/fneur.2024.1348439

**Published:** 2024-05-02

**Authors:** Anandhan Dhanasingh, Stefan Bryde Nielsen, Fabrice Beal, Soeren Schilp, Roland Hessler, Claude Jolly, Ingeborg Hochmair

**Affiliations:** Research and Development Department, MED-EL GmbH, Innsbruck, Austria

**Keywords:** cochlear implant, straight electrode, pre-curved electrode, electrical stimulation, trauma

## Abstract

The optimal placement of a cochlear implant (CI) electrode inside the scala tympani compartment to create an effective electrode–neural interface is the base for a successful CI treatment. The characteristics of an effective electrode design include (a) electrode matching every possible variation in the inner ear size, shape, and anatomy, (b) electrically covering most of the neuronal elements, and (c) preserving intra-cochlear structures, even in non-hearing preservation surgeries. Flexible electrode arrays of various lengths are required to reach an angular insertion depth of 680° to which neuronal cell bodies are angularly distributed and to minimize the rate of electrode scalar deviation. At the time of writing this article, the current scientific evidence indicates that straight lateral wall electrode outperforms perimodiolar electrode by preventing electrode tip fold-over and scalar deviation. Most of the available literature on electrode insertion depth and hearing outcomes supports the practice of physically placing an electrode to cover both the basal and middle turns of the cochlea. This is only achievable with longer straight lateral wall electrodes as single-sized and pre-shaped perimodiolar electrodes have limitations in reaching beyond the basal turn of the cochlea and in offering consistent modiolar hugging placement in every cochlea. For malformed inner ear anatomies that lack a central modiolar trunk, the perimodiolar electrode is not an effective electrode choice. Most of the literature has failed to demonstrate superiority in hearing outcomes when comparing perimodiolar electrodes with straight lateral wall electrodes from single CI manufacturers. In summary, flexible and straight lateral wall electrode type is reported to be gentle to intra-cochlear structures and has the potential to electrically stimulate most of the neuronal elements, which are necessary in bringing full benefit of the CI device to recipients.

## Introduction

The World Health Organization estimates that approximately 430 million individuals with hearing loss require treatment (1). Cochlear implants (CIs) are a widely accepted treatment option for those whose hearing threshold is ≥70 dB, which is considered severe-to-profound sensorineural hearing loss (2). A CI consists of an external audio processor and implantable components. The external audio processor receives sound signals and converts them to digital signals based on sound frequencies, which are then applied to the electronics in the hermetic case through an inductive link. The electronics in the hermetic case convert the digital signal to electrical impulses and send them to the scala tympani (ST) compartment of the cochlea through the electrode. Electrical stimulation from the ST reaches the ganglion cell bodies in Rosenthal’s canal through the peripheral neural fiber-endings at the organ of Corti, which are further transferred to the auditory cortex where it is perceived as sound (3).

The success of cochlear implantation depends on several factors. A good match between the CI electrode and cochlear anatomy covers most neuronal elements with electrical stimulation in creating an effective electrode–neural interface (ENI), making it a device-related factor (4). Different surgical techniques practiced by operating surgeons aim for ST electrode placement, ideally causing zero trauma to the intra-cochlear structures, making it a surgery-related factor (5). Patient-related factors that are reported to affect CI outcomes include the state of preoperative hearing, etiology of hearing loss, age at implantation, deafness duration, pre-lingual versus post-lingual hearing loss, adaptation to electrical stimulation by the recipient’s brain, and motivation of the recipient to use the speech processor regularly (6).

Optimal placement of the electrode inside the cochlea requires a combination of an effective electrode design and superior surgical skills. While surgical skills can be improved through continuous education, an electrode design is the result of a profound scientific understanding of the mechanical behavior of the electrode and the cochlear micro-anatomy. The electrode design and philosophy differ significantly among the CI manufacturers in terms of its (a) length, (b) shape configuration (straight versus pre-shaped), (c) number of stimulating channels, (d) space between stimulating channels, (e) distribution of metal wires to every stimulating channel, (f) shape of the channel exposed, and (g) tip geometry. According to Bierer (7), the electrode–neural interface (ENI) electrically excites a certain population of nerve fibers by placing an electrode channel in proximity. This can be affected by the current flow, channel interaction, electrode position, electrode misplacement, spiral ganglion loss, and channel independence.

## Structure of this review article

This review article on CI electrode design covers (i) different stages of multichannel electrode design per manufacturer, (ii) electrode portfolio as of 2024 from all four CI manufacturers, (iii) morphological variations in the inner ear and electrode choices, (iv) distribution of neuronal cell bodies and effective electrode insertion depth on hearing outcomes, (v) influence of electrode design on intra-cochlear delicate structures, (vi) electrode array migration and preventive solution, (vii) reports on incomplete insertion of electrode arrays, (viii) comparison of hearing performance between pre-curved vs. straight electrode from single CI manufacturers, (ix) literature on electrode type affecting facial nerve stimulation with CI, (x) electrode choice for cases with intra-cochlear tissue occlusion, and (xi) effect of electrode insertion approach on intra-cochlear tissue formation. A literature review of articles was conducted in analyzing the angular electrode insertion depth by different electrode arrays and its effect on hearing outcomes. Three-dimensional (3D) images of the inner ear were prepared using a freeware 3D slicer^1^ as previously described (8).

### Different stages of multichannel electrode design per manufacturer

A CI *per se* does not restore hearing in individuals with deafness. Instead, it provides frequency-specific electrical stimulation by placing an electrode covering most neuronal cell bodies. The first multichannel CI developed by MED-EL was implemented in 1978. The electrode had eight platinum contact pads that were opened on both sides and separated by equal distances. It is a 30 mm-long straight electrode (9). In 1981, Cochlear^®^ introduced the Nucleus 22 device with 22 evenly spaced full-band platinum ring electrodes. This electrode was designed to be 25 mm in length in a straight configuration to stimulate the cochlear basal turn (10). In 1987, Advanced Bionics introduced the Clarion C1 device, which had the first pre-curved electrode aimed at perimodiolar placement with 16 spherical (ball) contacts distributed within 25 mm to stimulate the cochlear basal turn (11). This pre-curved electrode needed a stiff tube made of Teflon® to make it straight before the insertion inside the cochlea. The stiff tube required to be pulled back to bring the electrode closer to the modiolus wall (10). These were first-generation electrode variants developed by three major CI companies on an experimental basis.

With a better understanding of how these first-generation electrodes provide hearing benefits to patients, MED-EL increased the number of stimulation channels from 8 to 12. This electrode was fabricated with a length of 31.5 mm to cover the entire frequency range physically. This electrode was marketed in 1996 with the commercial name “STANDARD” electrode array (12). In 2000, Advanced Bionics explored perimodiolar placement from a straight electrode design by employing a silastic dummy element to push the electrode contacts closer to the modiolar trunk (13). Unfortunately, a silastic dummy element parallel to the electrode increased the risk of meningitis and was removed from the market as a precautionary measure (14). Cochlear^®^ introduced a perimodiolar electrode concept (Contour) to attach the modiolus wall mainly to the cochlear basal turn. This electrode employs a platinum-made stylet wire for straightening before implantation (15). Around this time, MED-EL continued expanding its electrode variants and added “MEDIUM (24 mm)” and the “COMPRESSED (16 mm)” electrodes to its portfolio, to match the abnormal inner ear anatomies. In 2003, Advanced Bionics introduced a 22 mm-long straight electrode to achieve basal coverage (360° angular insertion depth [AID]) (16). It was named 1J as the distal end of the electrode had a pre-shaped configuration resembling the letter “J” (17). The very first version of flexible electrode design from MED-EL was introduced in 2004 under the electrode variant name “FLEX” and with a length of 31.5 mm. This is commercially called the FLEX SOFT, which has five apical channels opened on one side compared to both sides in the STANDARD electrode (18).

Advanced Bionics developed the “HELIX” electrode in 2005, which is a perimodiolar electrode type to cover the basal turn of the cochlea (360° of AID) (16). Around this time, Cochlear^®^ already introduced its second-generation perimodiolar electrode (Contour Advance), fine-tuning its first-generation electrode by modifying the rounded tip to a soft conical tip (19).

Until 2020, MED-EL continued to expand its FLEX electrode portfolio to lengths of 24, 28, 20, and 26 mm to match all cochlear size variations and cochleae with low-frequency residual hearing (20). Cochlear^®^ introduced a 15 mm-long straight configuration electrode in 2011 to support the concept of electric acoustic stimulation (21). In 2012, Cochlear^®^ introduced “Slim Straight,” a 20–25 mm-long electrode (between the electrode tip and the first marker–second marker at the proximal end) (22). This electrode has a stiffening element at the transition of electrode lead and the electrode array. Neurelec, later (in 2015) called Oticon currently acquired by Cochlear, introduced a 26 mm-long straight electrode (Digisonic^®^ SP) in 2004 (23). In 2006, Oticon fine-tuned the Digisonic^®^ SP electrode by reducing its length to 25 mm and commercially named it “Digisonic^®^ SP EVO.” In 2006, a new CI manufacturer from China (Nurotron) introduced a 22 mm-long electrode (24).

In 2013, Advanced Bionics developed another perimodiolar electrode (Mid-Scala), with the commercial claim that this electrode was designed to be positioned in the mid-ST, avoiding any contact with cochlear structures (25). In 2016, a slimmer version of the Contour Advance electrode was introduced by Cochlear^®^ called the “Slim Modiolar” electrode, which was 1 mm shorter than its predecessor (26). The Slim Modiolar electrode functions without any stylet wire, but with a polymer tube/sheath as a straightener, like the Clarion device from Advanced Bionics. A 23 mm-long straight electrode (Slim J) by Advanced Bionics was introduced in 2017, which is the slimmer version of its previous-generation electrode, the 1J electrode (27). In 2021, a 20 mm-long straight electrode (Slim20) was introduced by Cochlear^®^, which is identical to Slim Straight but without the 2nd white marker (28). The longest electrode (FLEX34) of industry, 34 mm in length, was introduced in 2023 by MED-EL to match larger sized cochlea (29).

### Current electrode portfolio from all four CI manufacturers

Within the straight lateral wall electrode type, 20 variants were identified from four different CI manufacturers, varying in length from 15 to 34 mm (Table 1).

**Table 1:** List of straight lateral wall electrodes.

CI manufacturer	Array variant	Array length (mm)	Market introduction
Advanced Bionics LLC	1J	22	2003
	Slim J	23	2017
Cochlear Corporation	Straight	25	1981
	Hybrid	15	2011
	Slim Straight	20 (up to the first marker) and 25 (up to the second marker)	2012
	Slim 20	20	2021
MED-EL GmbH	STANDARD	31.5	1996
	COMPRESSED	15	2000
	MEDIUM	24	2002
	FLEX SOFT	31.5	2004
	FLEX 24	24	2004
	FLEX 28	28	2011
	FLEX 20	20	2013
	FORM 19	20	2013
	FORM 24	24	2013
	FLEX 26	26	2018
	FLEX 34	34	2023
Nurotron	STANDARD	22	2006

Within the perimodiolar electrode type, only three variants were from two CI manufacturers, ranging in length from 17.5 mm to 18.5 mm (Table 2).

**Table 2:** List of perimodiolar electrodes.

CI manufacturer	Array variant	Array length (mm)	Market introduction
Advanced Bionics LLC	Mid-Scala	18.5	2013
Cochlear Corporation	Contour Advance	18.5	2005
	Slim Modiolar	17.5	2016

With all four CI manufacturers offering the straight lateral wall electrode type, the straight lateral wall electrode type may have a commercial demand along with reported lower electrode insertion complications, according to the latest published systematic literature review (30). Nevertheless, the perimodiolar electrode design is useful in cases such as otosclerosis and fibrous tissue occlusion of the cochlea.

### Morphological variations in inner ear and electrode choices

The normal anatomy of the inner ear is characterized by 2½ cochlear turns on average, although 20–30% of children with congenital deafness have some degree of inner ear anatomical variations/malformations (31, 32).

**Figure 1:**
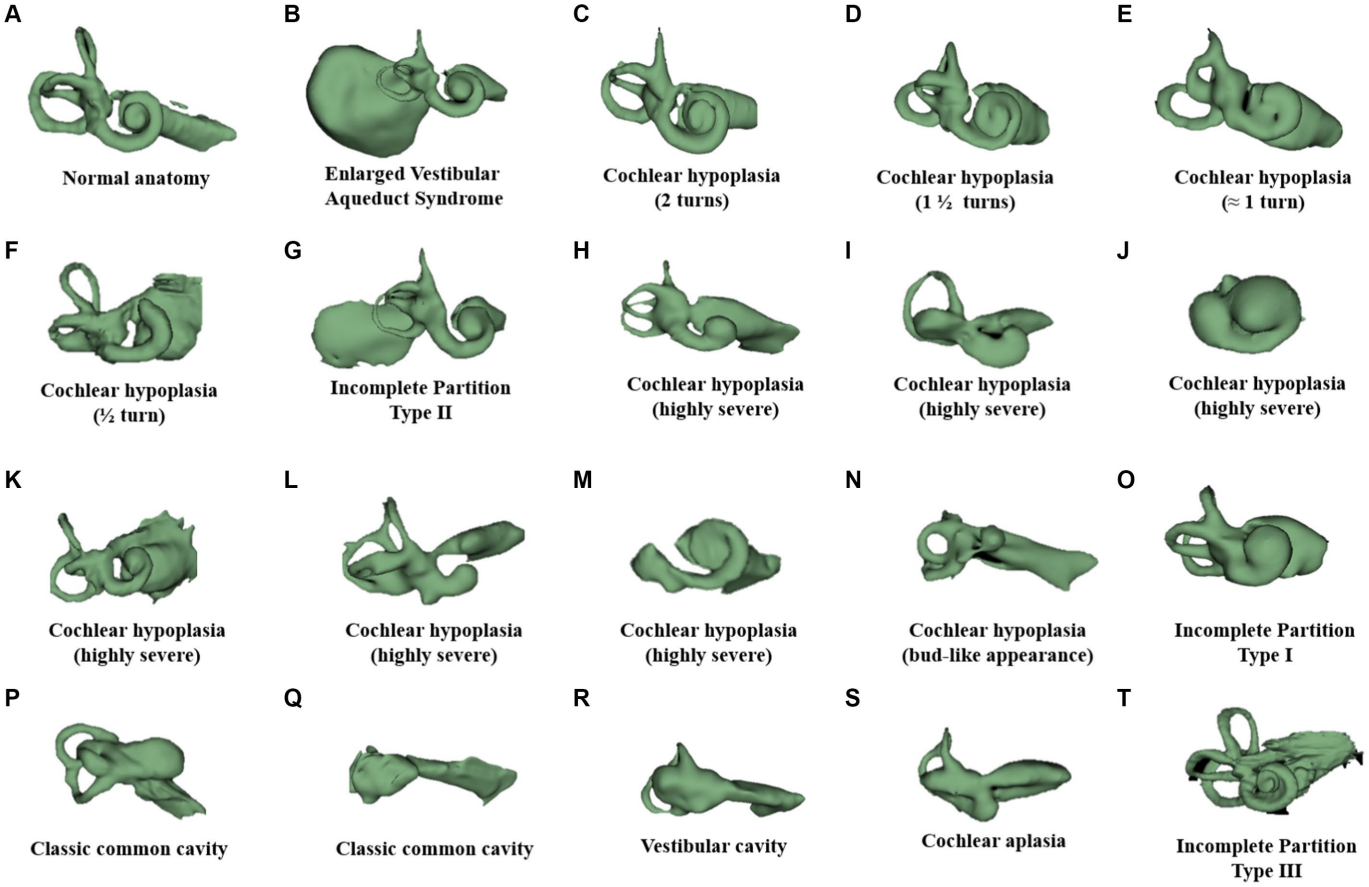
Anatomical variation of the human inner ear. Anonymized CT scans of temporal bones with different inner ear anatomies were kindly provided by St. Petersburg ENT and Speech Research Institute, Russia (33). **(A)** Normal anatomy; **(B)** almost normal cochlea anatomy but with an enlarged vestibular sac; cochlear hypoplasia with **(C)** 2 turns of the cochlear lumen; **(D)** 1½ turns of the cochlear lumen; **(E)** approximately 1 turn of the cochlear lumen; **(F)** only ½ turn of the cochlear lumen; **(G)** incomplete partition type II with enlarged vestibular aqueduct sac; **(H–M)** severe form of cochlear hypoplasia; **(N)** cochlear hypoplasia with bud-like cochlea; **(O)** incomplete partition type I; **(P,Q)** common cavity; **(R)** cochlear aplasia with vestibular cavity; **(S)** cochlear aplasia; and **(T)** incomplete partition type III.

Figure 1 illustrates a human inner ear with anatomical variations. A normal anatomy cochlea characterized by 2½ turns of cochlear lumen (Figure 1A) with profound hearing loss would require a full-coverage electrode (Tables 1, 2). However, whether those full-coverage electrodes from each CI manufacturer are physically long enough to sufficiently cover the majority of the cochlea remains unclear. Enlarged vestibular aqueduct syndrome characterized by a cochlear lumen that is almost available for 1½ turn leaving the apical portion cystic, along with an enlarged vestibular sac (Figure 1B), would require an electrode length of 24–28 mm that would cover approximately 540° of angular depth, depending on the cochlear size. Cochlear hypoplasia characterized with 2 turns (Figure 1C), 1½ turns (Figure 1D), 1 turn (Figure 1E), and ½ turn (Figure 1F) would need electrode arrays in medium (24 mm) to short lengths (15–20 mm). Incomplete partition (IP) type II (Figure 1G) characterized by the normal presence of a basal turn and cystic after along with an enlarged vestibular sac would require a medium-length electrode (24 mm) with a good sealing feature to prevent cerebrospinal fluid (CSF) gusher or oozing. A severe form of cochlear hypoplasia (Figures 1H–N) would typically need short-length electrodes (15–20 mm). IP type I (Figure 1O) characterized by the separation of the cystic cochlea from the vestibule and IP type III (Figure 1T) characterized by an absence of a central modiolus trunk and a wide internal auditory canal (IAC) would require a short electrode (≈20 mm), preferably with stimulating channels on both sides and good sealing feature to prevent CSF gusher. Cavity-type malformation (Figures 1P–R) characterized by a lack of central modiolus trunk requires the placement of an electrode with an appropriate length in a loopy format covering the circumference of the cavity where neuronal elements are distributed along the outer wall (34). In cochlear aplasia (Figure 1S), although placing an electrode in the vestibular portion is contraindicated as per the expert’s opinion (31), CI was still attempted with some success as per the report of Kim et al. (35).

**Figure 2:**
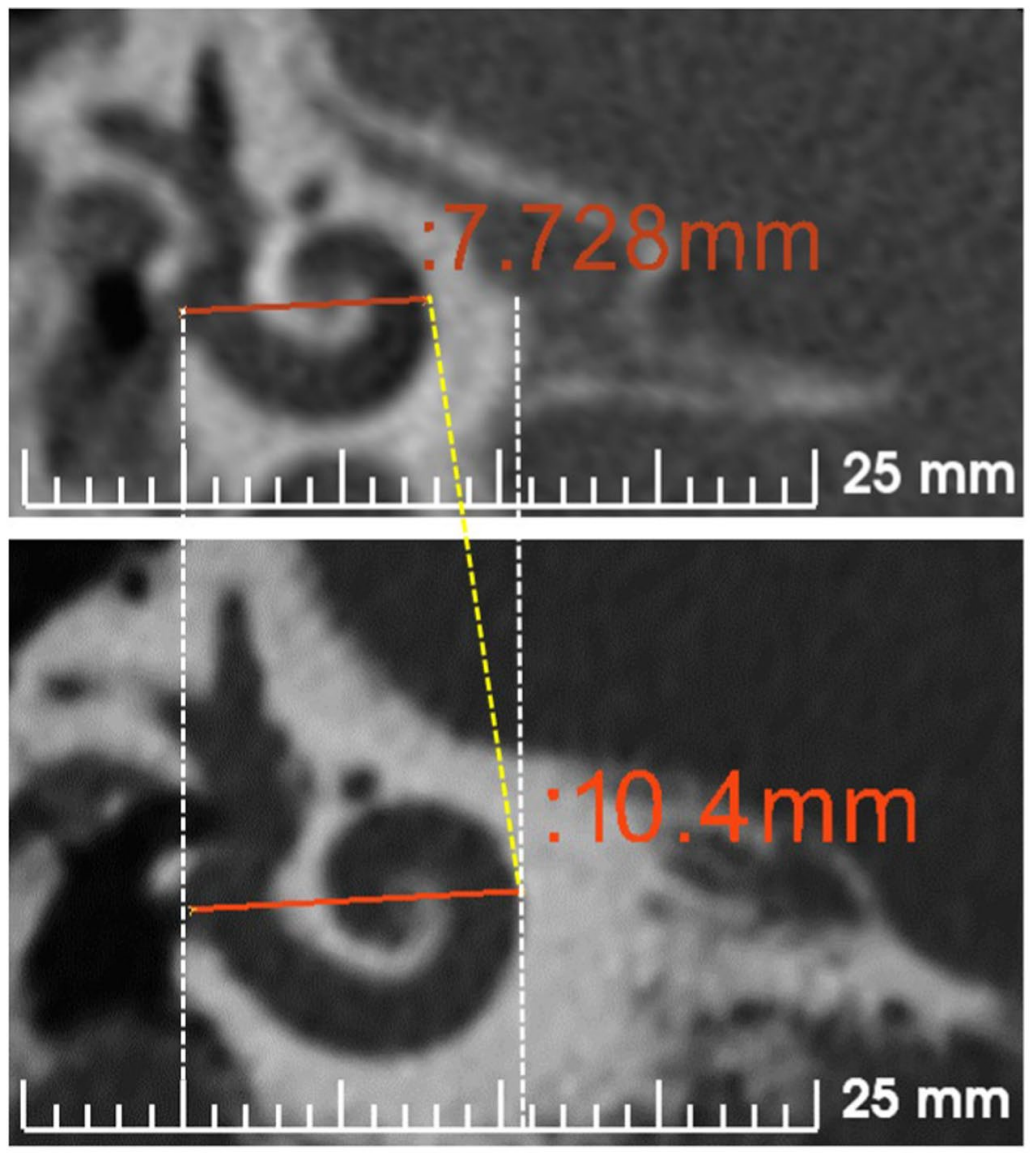
Cochlear size measured according to *A*-value in the oblique coronal view. CT scans showing the cochleae of different sizes are from anonymized subjects from St. Petersburg ENT and Speech Research Institute, Russia (33).

The normal anatomy of the inner ear varies in size (36). Figure 2 demonstrates two different sizes of the cochlea as measured by the cochlear basal turn diameter (*A*-value). The *A*-value is an indirect measure of cochlear duct length.

**Figure 3:**
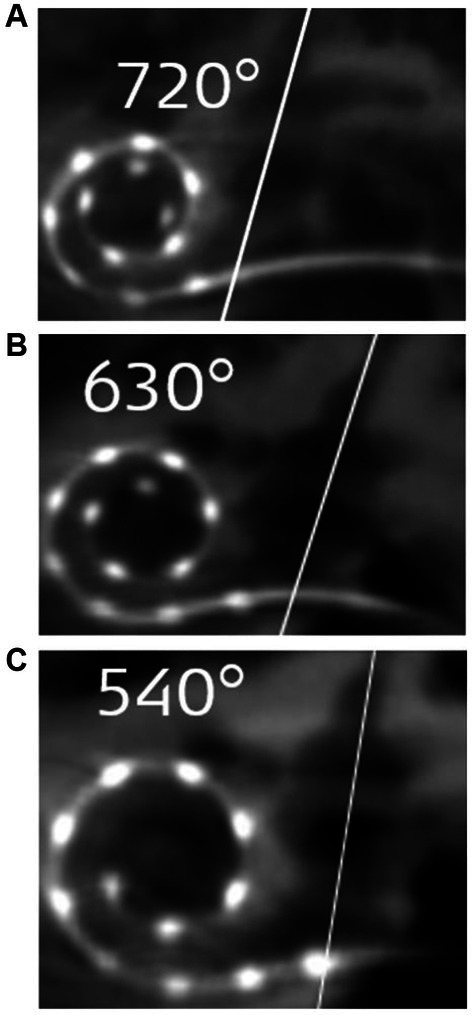
Postoperative images demonstrating STANDARD electrodes in three different sizes of cochleae (37). **(A)** An angular insertion depth (AID) of 720° in a regular-sized cochlea, **(B)** 630° of AID in a slightly bigger than regular sized cochlea, and **(C)** only 540° of AID in a significantly bigger sized cochlea. The white slanted line cutting through the electrode points to the cochlear entrance. Reproduced by permission of Williams and Wilkins Co.

Although assessing the preoperative images for cochlear size measurement is an effort, considering the concept that one length electrode would fit every cochlea will result in different insertion depths in the cochlea of different sizes (Figures 3A–C).

**Figure 4:**
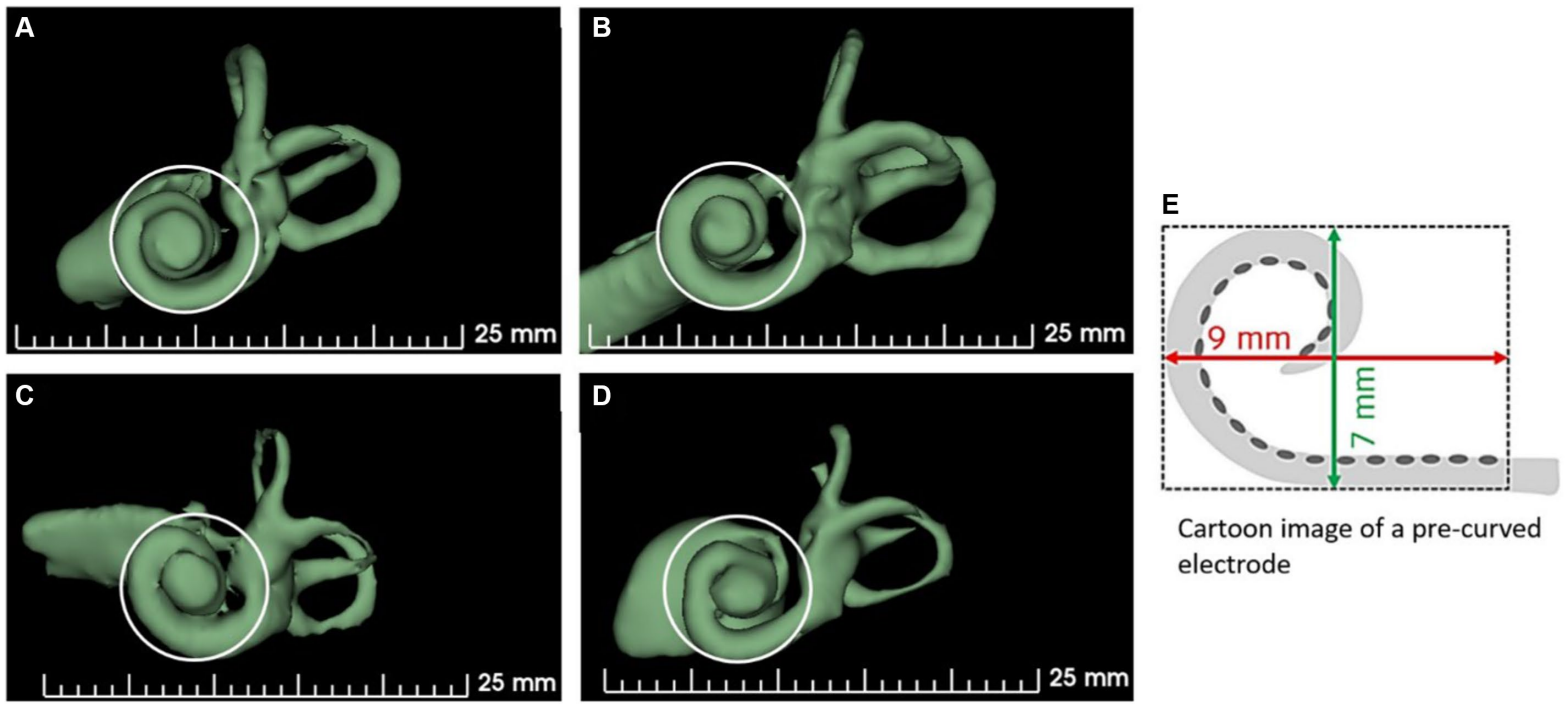
Shape variations in the cochlear basal turn. **(A)** More circular, **(B)** elliptical, **(C)** extended elliptical-shaped, **(D)** triangular, and **(E)** a perimodiolar electrode with a fixed size and shape. 3D segmented inner ear from CT scans of cochleae with varying shapes of basal turn are from anonymized subjects from St. Petersburg ENT and Speech Research Institute, Russia (33).

The shape of the cochlear basal turn is another important morphological variation that has not been studied in detail in cochlear implantation. Khurayzi et al. (38) have reported that the ratio between the width and length of the basal turn ≥0.75 indicates a round cochlear basal turn, which would otherwise indicate an elliptical basal turn. Rask-Andersen et al. (39) have reported overall variations in cochlear morphology using corrosion-cast models of normal cochleae without analyzing basal turn shape variations. Figure 4 demonstrates cochlear samples identified with different shapes of the cochlear basal turn [more circular shape (A), elliptical shape (B), extended elliptical shape (C), and triangular shape (D)]. Commercially available perimodiolar electrodes are fabricated with predetermined shapes, which may not match with every variation in the shape of the basal turn, making it difficult to offer consistent placement close to the modiolus in every case. However, the pullback technique as reported by Todt et al. (40) seems to bring the electrode close to the modiolus wall.

### Distribution of neuronal cell bodies and effective electrode insertion depth

**Figure 5:**
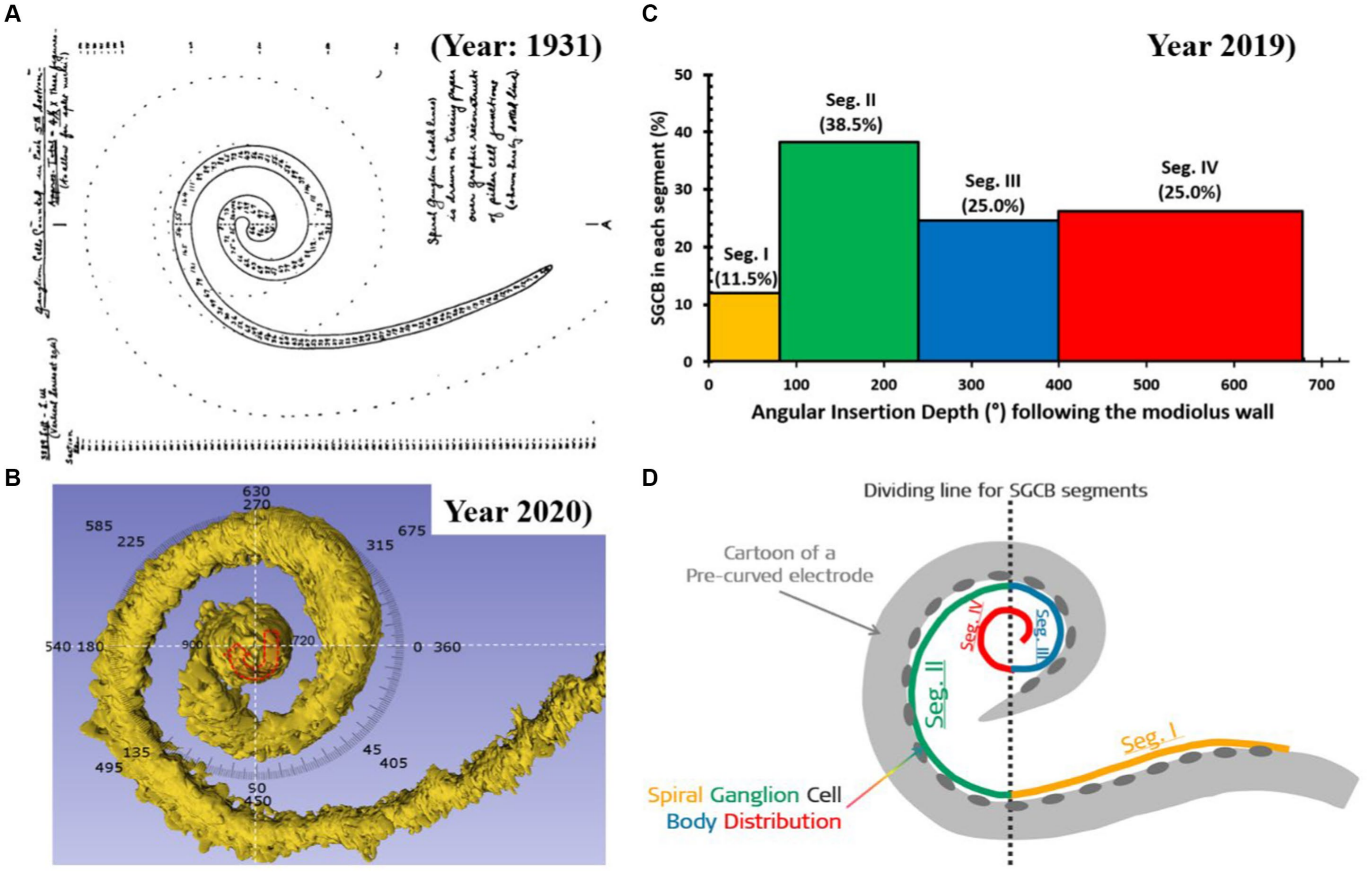
Distribution of spiral ganglion cell bodies (SGCBs) (40). **(A)** Outline of Rosenthal’s canal housing SGCBs; **(B)** 3D segmentation of SGCBs from the synchrotron phase-contrast image demonstrating its presence up to 680°–720° (Source: Courtesy of Dr. Hao Li and Dr. Helge Rask-Andersen, University Uppsala, Sweden, and Prof. Hanif Ladak and Dr. Sumit Agrawal, Auditory Biophysics Laboratory, Western University, London, in Ontario, Canada); **(C)** percentage of SGCBs in different segments of the cochlea; and **(D)** cartoon version of a perimodiolar electrode unable to cover segment 4 with electrical stimulation.

The electrical stimulus from the CI electrode delivered to the cochlea flows in the least path of resistance to reach the ganglion cell bodies. While the OC is known to extend until the helicotrema, spiral ganglion cell bodies (SGCBs) are distributed up to approximately 680°. From 1931 to 2023, 20 peer-reviewed publications have reported the angular distribution of SGCBs up to approximately 680° (41–43). Figure 5A presents an outline of Rosenthal’s Canal housing SGCBs, which are distributed up to 680°, as published in 1931. In 2020, with better imaging technology, SGCBs were 3D reconstructed, and their distribution was reconfirmed up to 680° (Figure 5B) (42). Individuals with normal hearing have been reported to have 33,000 SGCBs on average, covering both the basal and middle turns of the cochlea. Beyond an angular depth of 400°, the SGCBs were approximately 7,200 (41) (Figure 5C). The perimodiolar electrode was not sufficiently long to electrically stimulate this segment of the cochlea (Figure 5D). However, a longer length straight lateral wall electrode does cover segment IV of the cochlea, which results in a greater number of electrically stimulated SGCBs.

**Figure 6:**
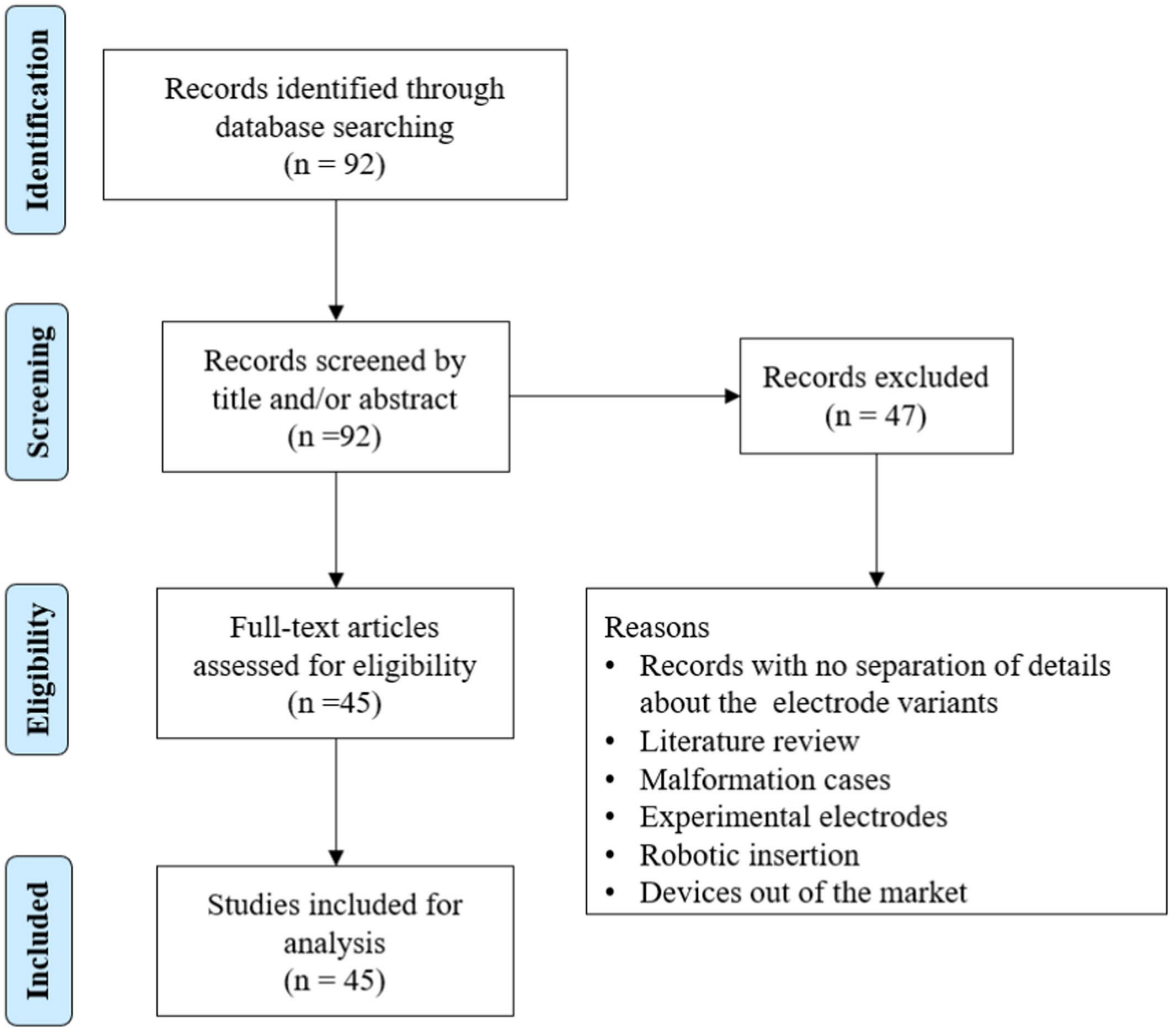
Literature review of articles reporting on electrode angular insertion depth.

To understand the AID offered by different electrode variants in patient cases, we searched the PubMed database using the term “angular insertion depth of cochlear implant electrode” (Figure 6).

In total, 92 articles were identified from the initial search, of which 45 articles reported on AID with electrode variants. The results are summarized in Table 3. A medium-length electrode (≈24 mm) from any CI manufacturer would reach an average angular depth of 406°. A perimodiolar electrode from any CI manufacturer would cover an average angular depth of ≤394°. A straight lateral wall electrode type of lengths 28 mm and 31.5 mm would reach an average AID of 546° and 625°, respectively.

**Table 3:** Review of literature on angular insertion depth by different electrode variants (references in Supplementary material S1).

No.	Study	Electrode type	No. of cases analyzed	Angular insertion depth (°)			
				Pre-shaped	STANDARD/FLEX SOFT	FLEX28	FLEX24/SS/SJ
1	Canfarotta et al. (2022)	Straight	75	—	628	571	—
2	Thimsen et al. (2022)	Straight	19	—	663	581	—
3	Razmovski et al. (2022)	Perimodiolar/straight	13/23	402	—	—	389
4	Fan et al. (2022)	Straight	29	—	—	—	332
5	Andersen et al. (2022)	Perimodiolar/straight	32	399		497	439
6	Högerle et al. (2021)	Straight	378	—	640	—	—
7	Lee et al. (2021)	Perimodiolar	95	375	—	—	—
8	Speigel et al. (2021)	Straight	108	—	615	525	—
9	Goehring et al. (2021)	Perimodiolar (MS)	8	—	—		—
10	Labadie et al. (2021)	Straight	1	—	—	539	—
11	Morrel et al. (2021)	Perimodiolar	6	344			—
12	Canfarotta et al. (2021)	Straight	51	—	641	—	—
13	Heutink et al. (2021)	Perimodiolar/straight	129	386	—	—	347
14	Canfarotta et al. (2021)	Straight	19	—	620	—	423
15	Canfarotta et al. (2021)	Straight/perimodiolar	50	364, 400 (MS)	641	509	400 (F24)
16	Lenarz et al. (2020)	Straight	20	—	—	—	393 (SJ)
17	Khan et al. (2020)	Straight	86	—	600	510	400 (F24, SS, SJ)
18	Canfarotta et al. (2020)	Straight	13	—	620	—	423 (M)
19	Noble et al. (2020)	Perimodiolar	57	381	—	—	—
20	Canfarotta et al. (2020)	Straight	111	—	636	558	428 (F24)
21	Canfarotta et al. (2020)	Straight	48	—	630	570	464 (F24)
22	Nassiri et al. (2020)	Perimodiolar	24	388	—	—	—
23	Rivas et al. (2019)	Straight	40	—	—	—	416 (SJ)
24	Rathgeb et al. (2019)	Straight	50	—	—	512	—
25	Canfarotta et al. (2019)	Straight	20	—	619	578	422 (F24)
26	Abd El Aziz et al. (2019)	Perimodiolar	20	—	—	—	—
27	Yamamoto et al. (2019)	Perimodiolar	57	354	—	518	—
28	An et al. (2018)	Straight	21	—	—	562	451 (SS)
29	Skarzynski et al. (2018)	Straight	54	—	—	—	375
30	Dietz et al. (2018)	Straight	11	—	—	—	368
31	Iso-Mustajärvi et al. (2017)	Perimodiolar	20	400	—	—	—
32	O’Connell et al. (2017)	Straight	48	—	584	575	408
33	van der Jagt et al. (2016)	Perimodiolar (MS)	96	424	—	—	—
34	Roy et al. (2016)	Straight	25	—	584	—	389
35	Svrakic et al. (2016)	Perimodiolar (MS)	63	389	—	—	—
36	Bengalem et al. (2016)	Perimodiolar (MS)	7	435	—	—	—
37	O’Connell et al. (2016)	Perimodiolar (MS)	129	384, 393 (MS)	—	—	—
38	Nordfalk et al. (2016)	Straight	39	—	628	593	507 (F24)
39	Frisch et al. (2015)	Perimodiolar (MS)	8	436	—	—	—
40	Skarżyński et al. (2014)	Straight	55	—	—	—	424
41	Schatzer et al. (2014)	Straight	7	—	610	—	331 (M)
42	Pearl et al. (2013)	Straight	17	—	592	—	—
43	Trieger et al. (2011)	Straight/perimodiolar	15	469	700	—	—
44	Radeloff et al. (2008)	Straight/perimodiolar	46	—	—	—	—
45	Xu et al. (2000)	Perimodiolar	7	335	—	—	—
	**Total/mean ± std. dev**		**2,214**	**394 ± 36**	**625 ± 28**	**546 ± 53**	**406 ± 42**

M, medium; MS, Mid-Scala; F24, FLEX24; SS, Slim Straight; SJ, Slim J.

**Figure 7:**
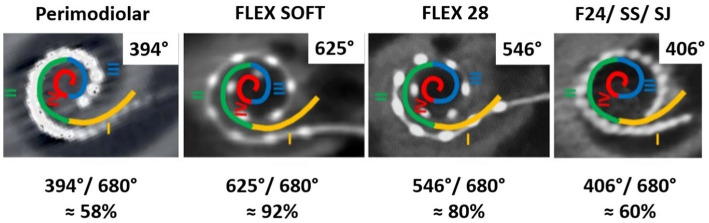
Neuronal coverage by electrode variants.

Considering the 680° of angular depth to which the SGCBs are distributed, a perimodiolar electrode would provide approximately 58% of neural coverage, whereas a medium-length (≈24 mm) electrode would provide approximately 60% of neural coverage. When fully inserted, the FLEX28 and FLEX SOFT electrodes provided approximately 80 and 92% neural coverage, respectively, as shown in Figure 7. All these AID and percentages of neural coverage are mere numbers and carry value if they influence hearing outcomes in CI recipients.

**Figure 8:**
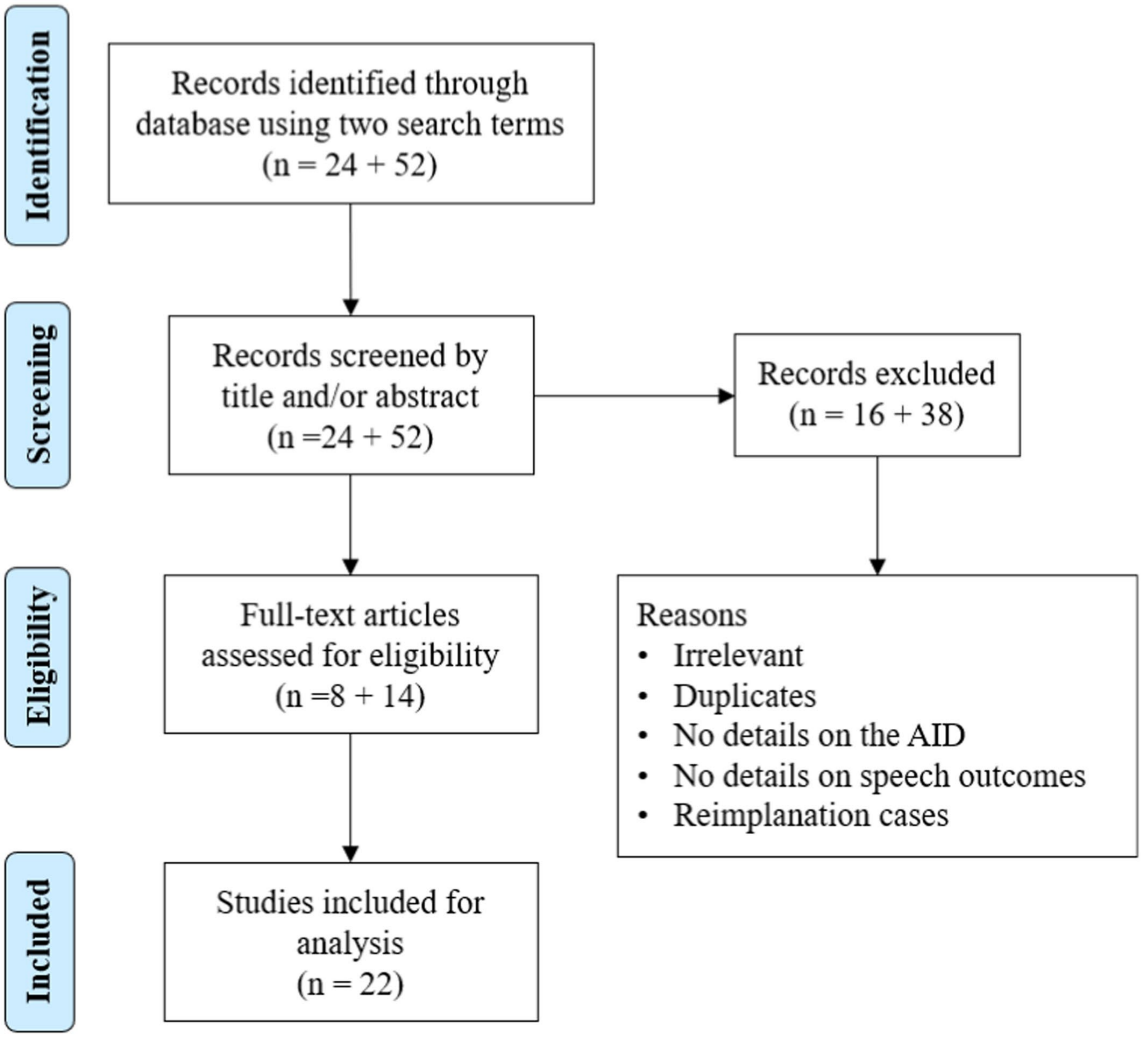
Literature review process of studies on electrode angular insertion depth and associated hearing outcomes.

To determine whether the AID of electrode has been reported to influence postoperative hearing outcomes with CI, the literature was reviewed using the search terms “cochlear implant electrode insertion depth and speech recognition scores” and “cochlear implant electrode insertion depth and hearing scores” in PubMed (Figure 8).

In total, 76 articles were identified in the initial search using two search terms, of which 22 reported an association between AID and hearing outcomes. The results are summarized in Table 4. Of the 22 articles, 15 reported a positive correlation, 2 reported a negative correlation, and the remaining 5 reported no correlation between the two variables. In those two papers reporting a negative correlation, the electrodes used mainly covered the cochlear basal turn but not the middle turn. Seven other articles were identified outside the search terms for electrode AID and hearing outcomes (Table 5). Of seven articles, six reported a positive correlation and one article reported a negative correlation between electrode AID and hearing outcomes.

**Table 4:** Reports identified using the search terms on correlation between electrode angular insertion depth and hearing outcomes (references in Supplementary material S2).

No.	Studies	Region of origin	Electrode variant	Angular insertion depth (mm/AID°)	Correlation between AID and hearing outcomes
1	Alothman et al. (2023)	Saudi Arabia	FO24 vs. F28	460°–530°	Positive
2	Canfarotta et al. (2022)	USA	F28 vs. FS	571°–628°	Positive
3	Fan et al. (2022)	China	Shanghai Lishengte LCI20PI	240°–500°	Positive
4	Lo Rosso et al. (2022)	Italy	F28, F24, CA, SM, SS, MS, SJ, EVO, CLA	—	No significance
5	Heutink et al. (2021)	The Netherlands	SS vs. CA	347.6°–386.2°	Positive
6	Canfarotta et al. (2020)	USA	MED and STD	460°–720°	Positive
7	Canfarotta et al. (2020)	USA	F24, F28, FS	428°–558°–636°	Positive
8	Nassiri et al. (2020)	USA	SM	360°–450°	Positive
9	Selleck et al. (2019)	USA	All three FDA-approved CI devices	—	Positive
10	Chakravorti et al. (2019)	USA	All three FDA-approved CI devices	376° (pre-curved)	Positive
				453° (straight)	(Among straight electrodes)
11	O’Connell et al. (2017)	USA	F24, F28, STD	408°–575°–584°	Positive
12	Hilly et al. (2016)	Canada	1J	<360°– > 360°	Positive
13	De Seta et al. (2016)	France	STD	403° (partial insertion); 643° (full insertion)	No significance
14	Nayak et al. (2016)	India	CI24RE, CA, 1J, SS	<180° > 360°	No significance
15	Roy et al. (2016)	USA	STD, MED	389°–583°	Positive
16	van der Marel et al. (2015)	The Netherlands	HiFocus 1, 1J	497°–479°	No significance
17	Holden et al. (2013)	USA	HiFocus 1, 1J, Helix, C, CA	—	Negative
18	Lee et al. (2010)	USA	Nucleus 22	—	Positive
19	Finley et al. (2008)	USA	AB/Clarion devices	—	Negative
20	Khan et al. (2005)	USA	Nucleus 22/24, Ineraid, Clarion	—	No significance
21	Yukawa et al. (2004)	Japan	Nucleus CI22M and CI24	210°–580°	Positive
22	Skinner et al. (2002)	USA	Nucleus 22	12–26 mm	Positive

C, Contour; CA, Contour Advance; FO24, FORM24; F24, FLEX 24; F28, FLEX28; FS, FLEX SOFT; SM, Slim Modiolar; SS, Slim Straight; MS, Mid Scala; SJ, Slim J; STD, STANDARD; MED, MEDIUM.

**Table 5:** Reports identified outside the search terms on the correlation between electrode angular insertion depth and hearing outcomes (references in Supplementary material S3).

No.	Studies	Region of origin	Electrode variant	Angular insertion depth (mm/AID°)	Correlation between AID and hearing outcomes
1	Lyutenski et al. (2021)	Germany	MS to F28	360°–560°	Positive
2	Ketterer et al. (2021)	Germany	F24, F28, FS, CA, SS, SM	199°–794°	Negative
3	Helbig et al. (2018)	Germany	F28	350°–730°	Positive
4	Büchner et al. (2017)	Germany	F20-24-28	360°–480°–585°	Positive
5	O’ Connell et al. (2016)	USA	SS	290°–600°	Positive
6	Buchman et al. (2014)	USA	MED and STD	423°–657°	Positive
7	Esquia et al. (2013)	France	F24 and FS	251°–720°	Positive

CA, Contour Advance; F24, FLEX 24; F28, FLEX28; FS, FLEX SOFT; SM, Slim Modiolar; SS, Slim Straight; MS, Mid Scala; STD, STANDARD; MED, MEDIUM.

All studies listed in Tables 4, 5 had heterogeneities associated with different electrode types/variants with stimulation strategies from four different CI manufacturers, tested using different audiological tests conducted in different languages. However, three-fourths of these studies reported a positive correlation between the electrode AID and hearing outcomes, regardless of the electrode type. Broader frequency coverage, a larger number of intra-cochlear sites for electrical stimulation, closer matching of electrical with place-equivalent acoustic pitches, greater spatial separation between adjacent electrode contacts improving spectral resolution, and electrically recruiting a greater number of neuronal cell bodies were some of the possible reasons given for better hearing outcomes with deeper insertion.

### Influence of electrode design on intra-cochlear delicate structures

**Figure 9:**
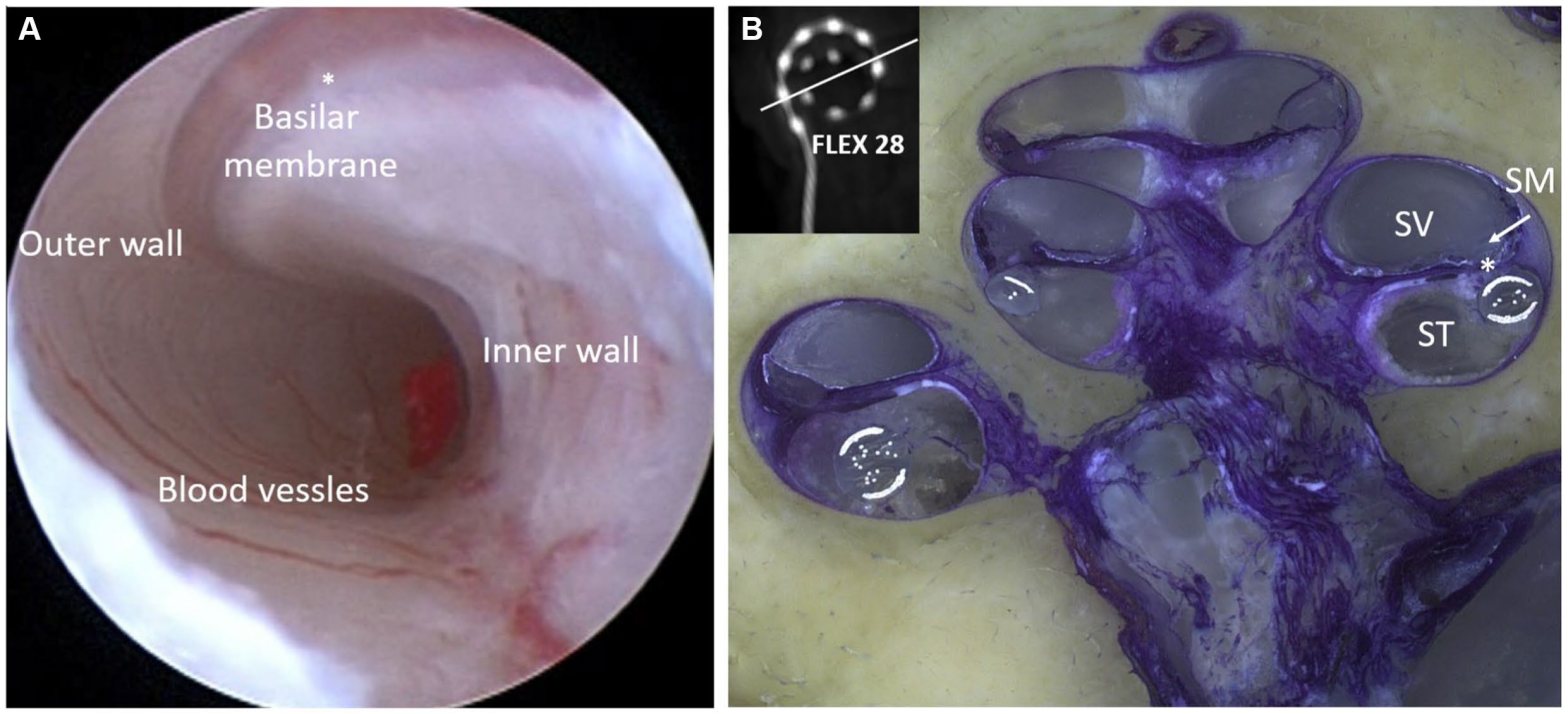
Closer look at the human cochlea. **(A)** Endoscopic view of scala tympani (ST) entering through the round window (RW) entrance demonstrating blood vessels on the floor, basilar membrane on the top, porous bony wall on the right, and smooth lateral wall on the left (reproduced by permission of Dr. Richard Chole, Washington, United States). **(B)** Histology image of the mid-modiolar section of the human cochlea with a 28 mm-long electrode placed fully inside the ST (image courtesy: Prof. Thomas Lenarz and Peter Erfurt from Hannover Medical School, Hannover, Germany).

The flexibility of an electrode array refers to its ability to accommodate the cochlear lumen with changing contours, avoid traumatizing delicate intra-cochlear structures, and achieve full insertion inside the cochlea. The preferred location for electrode placement of electrode was the ST compartment of the cochlea. Figure 9A demonstrates the endoscopic view of a live ST during the CI procedure. ST is like a tunnel lined with porous bone that forms the inner wall. The outer wall was significantly smoother along with any straight lateral wall electrode slide during insertion. The floor of the ST contains a series of blood vessels that are exposed to the surface. The basilar membrane is located at the top of the ST and separates the scala media from the ST. Structure preservation with minimal insertion trauma is the primary aim of every CI surgery. Figure 9B illustrates the full insertion of a 28 mm-long flexible electrode placed inside the ST.

**Figure 10:**
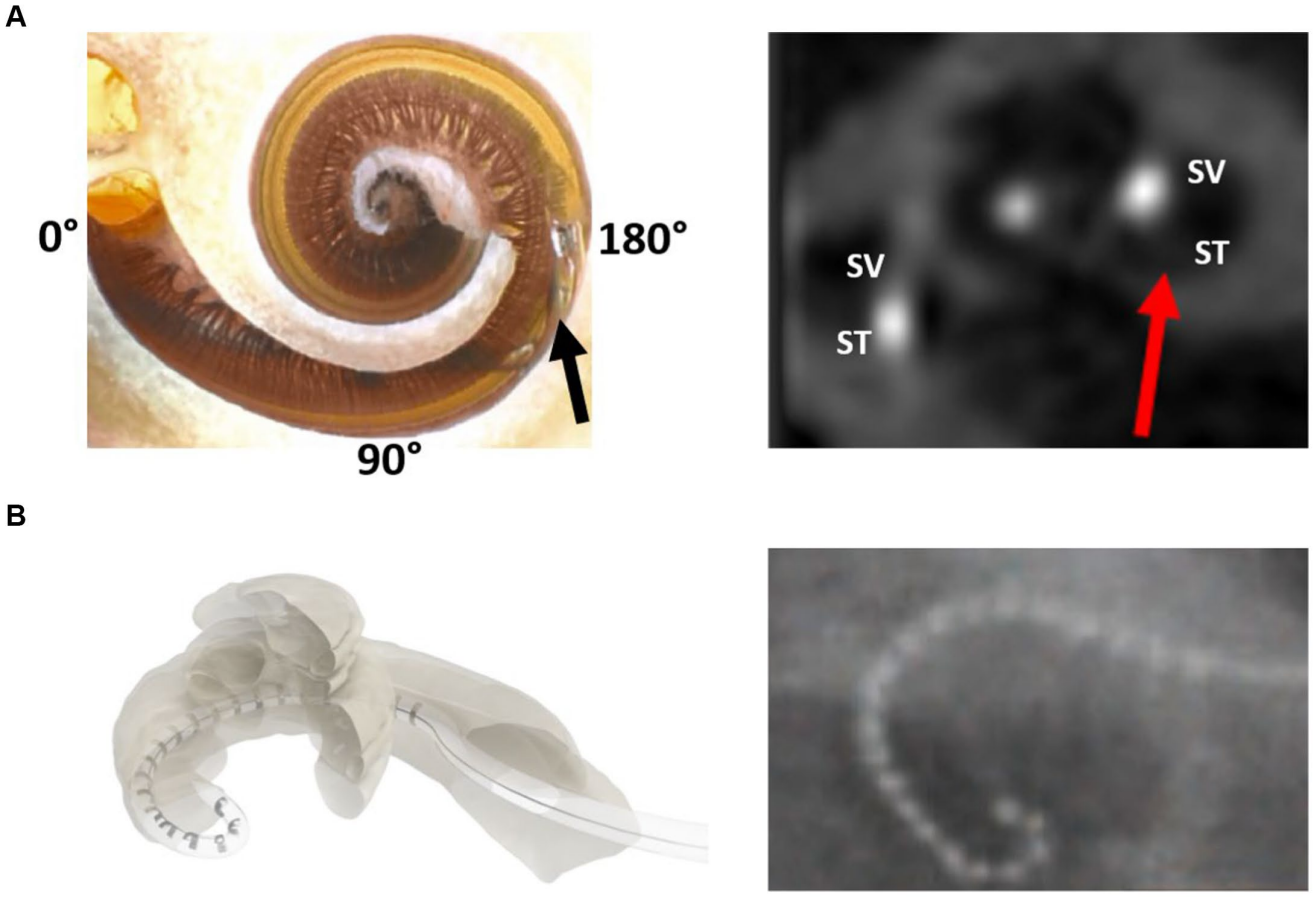
Illustration of electrode scalar deviation **(A)** and electrode tip fold-over **(B)** (30).

Electrode scalar deviation (ESD) is considered a severe form of intra-cochlear trauma as per Eshraghi (44) (Figure 10A). A recent systematic review of the literature on electrode design-related complications related to manual insertion of electrodes has reported that straight lateral wall electrodes have an 11% rate of ESD compared to styleted perimodiolar hugging electrodes (28.5%) (30). We analyzed the results [Table 3 of reference (30)] to determine the rate of ESD with the FLEX and Slim Modiolar electrodes as 4.8% (22 out of 453 FLEX electrode implantations) and 7.6% (28 out of 365 Slim Modiolar implantations), respectively. It has been widely reported that ESD results in lower hearing scores than ST placement (45, 46). The tip of the electrode folding over while inserting it inside the ST is referred to as the electrode tip fold-over (ETFO), which is another electrode design-related issue (Figure 10B). The same systematic review has reported an overall incidence rate of 5.4% for ETFO with perimodiolar electrode and only 0.5% for the straight lateral wall electrode type. Analysis of the results [Table 2 of reference (30)] to determine the incidence rate of ETFO with Slim Modiolar electrode revealed 6% (70 cases out of 1,158 implantations), which is slightly higher than that of the styleted perimodiolar electrode. Electrode array buckling or kinking inside the cochlea has been reported with an incidence rate of 2% in mixed-device types (47). Such buckling or kinking of the electrode can also lead to intra-cochlear structural damage.

### Electrode array migration and available solutions

**Figure 11:**
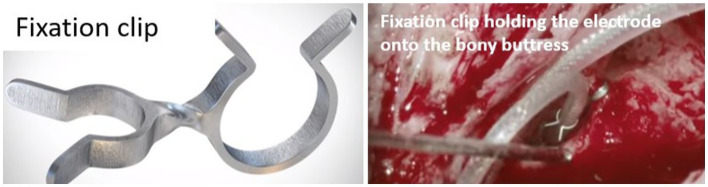
MED-EL fixation clip made of titanium. A surgical view of the fixation clip locking the electrode leads to the bony buttress of the middle ear.

The slipping of the electrode out of the cochlea immediately or after CI surgery is referred to as electrode array migration. The straight lateral wall electrode type has been reported to migrate at a rate of 3.2% compared to 0.5% for the perimodiolar electrode type (30). The perimodiolar electrode wraps around the inner wall, providing a natural fixation that minimizes the array migration rate. By contrast, the straight lateral wall electrode is gently positioned along the outer wall, which increases the rate of array migration when there is a disturbance to the excess electrode lead in the mastoid cavity. Recently, Goh et al. (48) reported that electrode migration is one of the common occurrences with straight electrodes and is more likely in implant recipients with obstructed or malformed cochleae. A fixation clip that locks the electrode lead to the middle ear structures (Figure 11) is one practical solution to minimize array migration with straight electrodes. Other surgical practices are locking the electrode lead in the undercuts on the skull, packing the mastoid cavity with bone pate, and drilling additional grooves in the facial recess to lock the electrode lead also referred to as the “Hannover technique.”

### Reports on incomplete insertion of electrode arrays

The incomplete insertion of an electrode array is undesirable. Mixed reports are available on this topic, with Nordfalk et al. (49) reporting an 18% failure rate to fully insert the FLEX28 and FLEX SOFT electrodes. Meanwhile, Canfarotta et al. (50) have reported a 0% failure rate to insert the FLEX SOFT electrode fully inside the cochlea. Ishiyama et al. (47) have reported that partial insertion of the electrode is highly associated in patients preoperatively diagnosed with difficult inner ear anatomy or occluded pathology, with an incidence rate of 1–2%. Lee et al. (51) conducted a histopathological analysis and reported that incomplete insertion is also influenced by the electrode encountering insertion resistance with the spiral ligament as was observed in 14 of 21 samples. Basal buckling is another electrode design-related issue mainly associated with the straight electrode, and this was reported with EVO^®^ from Oticon by Torres et al. (52) due to the sub-optimal insertion angle into the basal turn of the cochlea. A minimum of 8–10 stimulating channels inside the cochlea are needed to receive a meaningful performance gain with CI (53).

### Comparison between perimodiolar and straight electrode from single CI manufacturer

This section aims to bring together reports that have compared the overall performance of straight and perimodiolar electrodes from the same CI manufacturer to avoid any bias between manufacturers. One of the claimed advantages of a perimodiolar-placement electrode over a straight electrode is that it minimizes the intensity of stimulation, resulting in an increased battery life of the audio processor. In 2015, Jeong et al. (54) studied the battery consumption of an audio processor coupled to Contour Advance (perimodiolar) and Slim Straight (straight) electrodes implanted on each side in seven patients. They have reported that even though the intensity of electrical energy needed for auditory perception may be lower for the perimodiolar electrode than for the straight array, the dynamic range and battery consumption were similar.

Fitzgerald et al. (55), Doshi et al. (56), Garaycochea et al. (57), Sturm et al. (58), and MacPhail et al. (59) have reported that speech perception and frequency discrimination outcomes remain the same for both perimodiolar and straight electrode types from the same manufacturer. In 2021, Heutink et al. (60) reported better speech perception and frequency discrimination with a Contour Advance electrode than with a Slim Straight electrode. An in-depth review of the report by the authors of the current paper revealed that the Contour Advance electrode reached an AID of 386°, compared to the Slim Straight electrode with an AID of 347° only. One could argue that a higher insertion depth would electrically stimulate more neuronal elements, thereby contributing to better speech outcomes with the Contour Advance electrode. By contrast, Holder et al. (61) compared Slim Modiolar with Slim Straight electrodes in a matched cohort and reported favorable or similar results in terms of postoperative low-frequency pure-tone average, CNC scores, electrode impedance, and pulse duration in the Slim Modiolar electrode-implanted group compared to the Slim Straight electrode-implanted group from the same manufacturer.

In 2023, Patro et al. (62) compared a perimodiolar electrode type (Mid-Scala) with the straight lateral wall electrode type (Slim J) electrode of both electrode types from a single manufacturer to determine which electrode type is superior in terms of speech recognition and hearing preservation. The rate of ESD was 34.8% with the Mid-Scala compared to 16.1% with the Slim J electrode, resulting in a higher hearing preservation rate with the Slim J than the Mid-Scala electrode. Hearing scores in quiet and noisy conditions did not differ significantly between patients implanted with the two electrode types.

All the studies listed in this section had almost the same conclusion that speech recognition scores did not significantly differ between the electrode types, whereas perimodiolar electrodes had higher rates of ESD and ETFO, which need to be considered before selecting the electrode type for implantation.

### Literature on electrode type affecting facial nerve stimulation following CI treatment

Facial nerve stimulation is a post-activation complication of CI treatment. It is widely believed in the CI field that straight electrodes are more prone to facial nerve stimulation (FNS) than perimodiolar electrodes. In 2005, Smullen et al. (63) studied the rate of FNS by comparing the perimodiolar and straight electrodes implanted in their study cohort. Of the 600 patients with CI, 19 experienced FNS. The rate of FNS was similar (6.8%) between the perimodiolar and straight electrodes. In 2011, Berrettini et al. (64) have reported FNS in 11 of 119 patients with CI. Of the 11 patients with FNS, 10 were implanted with a perimodiolar electrode and only one with a straight electrode, both from the same CI manufacturer. In 2013, Seyyedi et al. (65) reported from a group of patients with otosclerosis, straight electrode (4 of 10) led to FNS, which was not the case with perimodiolar electrode (0 of 3), which favors perimodiolar electrode in such conditions. In 2020, Van Horn et al. (66), through a systematic literature review and meta-analysis, reported that straight electrodes implanted in patients with CI experienced FNS at a higher incidence rate (15.7%) than the perimodiolar electrode-implanted group (4.4%). All these reports reveal that FNS can occur with any electrode type, although its incidence is relatively lower with the perimodiolar electrode type. A recent systematic review by Alahmadi et al. (67) reports that modifying different fitting parameters successfully resolved FNS in 85.5% of the patients. Tri-phasic pulse stimulation in the MED-EL system (68) and pseudo-monophasic stimulation (69) in the Oticon system are typical examples of new fitting parameters that can minimize FNS.

### Intra-cochlear fibrosis and suitable electrode type

**Figure 12:**
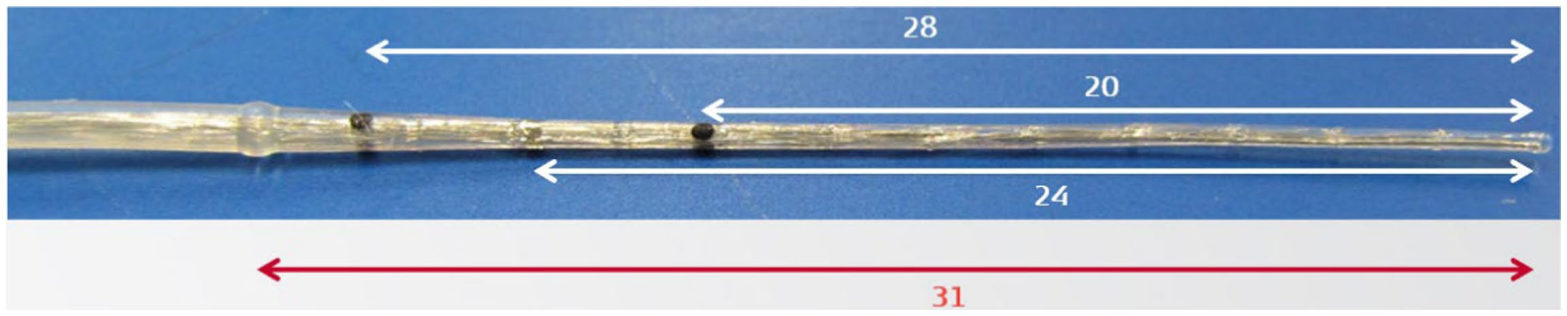
Stiff probe (dummy) device with insertion depth markers.

One of the advantages of the perimodiolar electrode, as observed in the field, is its application in cochleae with fibrotic occlusion. In such situations, a styleted perimodiolar electrode (Contour Advance, Mid-Scala) may assist in penetrating the fibrotic tissue. The stiff insertion dummy probe device from MED-EL is an alternative solution for dilating fibrotic occlusion (Figure 12). Recently, Hoffmann et al. (70) have reported the successful use of this stiff probe device in dilating the fibrous tissue-occluded cochlea in 33 cases, followed by the placement of a flexible straight electrode.

### Effects of electrode insertion approach on intra-cochlear tissue formation

Histopathological studies have reported that promontory bone-drilled cochleostomy (Coch) or extended round window (ERW) approaches lead to a greater amount of bone/fibrous tissue formation inside the cochlea around the electrode than the pure RW approach (71–73). Recently, Ketterer et al. (74) reported a reimplantation case in which explantation of the electrode from a failed CI was not possible because of neo-ossification covering the electrode, locking it inside the ST. This aspect forced the surgical team to cut off the electrode array to remain inside the ST and place the new electrode in the SV, again drilling a bony cochleostomy. Unfortunately, the perimodiolar electrode type requires either the ERW or Coch approach in most cases, whereas the straight electrode design is compatible with the RW approach for insertion.

## Conclusion

This review sheds light on the requirements of an effective CI electrode array design to match with overall variations reported in the size, shape, and anatomy of the human inner ear, as well as minimizing the intra-cochlear electrode insertion trauma. It is commonly agreed in the CI field that any degree of intra-cochlear trauma should be minimized during the electrode insertion process. Current scientific evidence indicates, at the time of writing this article, that the straight lateral wall electrode outperforms the perimodiolar electrode type by preventing electrode tip fold-over and scalar deviation. Most of the literature comparing the hearing performance of perimodiolar and straight electrode types from the same CI manufacturer did not show the superiority of one electrode type over the other. However, facial nerve stimulation is reported to be minimized with perimodiolar electrodes as compared to the straight electrode type, a problem that is solvable by triphasic pulse stimulation or pseudo-monophasic stimulation with the latter. Almost three-fourths of scientific reports evaluating the effect of electrode angular insertion depth on hearing outcomes confirm increased hearing benefits associated with electrical stimulation covering both basal and middle turns of the cochlea. This can be explained by facts like electrodes covering a broader frequency range, stimulating a larger number of neuronal cell bodies that are distributed up to 680° of angular insertion depth, greater spatial separation between adjacent electrode contacts, and closer matching of electrical with place-equivalent acoustic pitches.

## Author contributions

AD: Writing – review & editing, Writing – original draft, Methodology, Conceptualization. SN: Resources, Writing – review & editing. FB: Resources, Writing – review & editing. SS: Resources, Writing – review & editing. RH: Writing – review & editing, Resources. CJ: Writing – review & editing, Supervision. IH: Writing – review & editing, Supervision.

## Footnotes

^1^ https://www.slicer.org/

## References

[1] World Health Organization. (2021). WHO: 1 in 4 people projected to have hearing problems by 2050. Available at: https://www.who.int/news/item/02-03-2021-who-1-in-4-people-projected-to-have-hearing-problems-by-2050

[2] Zwolan TA Basura G. Determining cochlear implant candidacy in adults: limitations, expansions, and opportunities for improvement. Semin Hear. (2021) 42:331–41. doi: 10.1055/s-0041-1739283, PMID: 34912161 PMC8660165

[3] Dhanasingh A Hochmair I. Signal processing & audio processors. Acta Otolaryngol. (2021) 141:106–34. doi: 10.1080/00016489.2021.1888504 33818264

[4] Dhanasingh A Jolly C. An overview of cochlear implant electrode array designs. Hear Res. (2017) 356:93–103. doi: 10.1016/j.heares.2017.10.005, PMID: 29102129

[5] Friedland DR Runge-Samuelson C. Soft cochlear implantation: rationale for the surgical approach. Trends Amplif. (2009) 13:124–38. doi: 10.1177/1084713809336422, PMID: 19447766 PMC4111526

[6] Zhao EE Dornhoffer JR Loftus C Nguyen SA Meyer TA Dubno JR. Association of patient-related factors with adult cochlear implant speech recognition outcomes: a meta-analysis. JAMA Otolaryngol Head Neck Surg. (2020) 146:613–20. doi: 10.1001/jamaoto.2020.0662, PMID: 32407461 PMC7226297

[7] Bierer JA. Probing the electrode-neuron interface with focused cochlear implant stimulation. Trends Amplif. (2010) 14:84–95. doi: 10.1177/1084713810375249, PMID: 20724356 PMC4111350

[8] Dhanasingh A Dietz A Jolly C Roland P. Human inner-ear malformation types captured in 3D. J Int Adv Otol. (2019) 15:77–82. doi: 10.5152/iao.2019.6246, PMID: 31058598 PMC6483443

[9] Hochmair-Desoyer IJ Hochmair ES Burian K Fischer RE. Four years of experience with cochlear prostheses. Med Prog Technol. (1981) 8:107–19. PMID: 6895542

[10] Thomas Roland J. Jr. Huang Tina C. Fishman Andrew J.. Cochlear implant electrode history, choices, and insertion techniques. Available at: https://entokey.com/cochlear-implant-electrode-history-choices-and-insertion-techniques/

[11] Rebscher SJ Hetherington A Bonham B Wardrop P Whinney D Leake PA. Considerations for design of future cochlear implant electrode arrays: electrode array stiffness, size, and depth of insertion. J Rehabil Res Dev. (2008) 45:731–48. doi: 10.1682/JRRD.2007.08.0119, PMID: 18816423 PMC2562296

[12] Helms J Müller J Schön F Moser L Arnold W Janssen T. Evaluation of performance with the COMBI40 cochlear implant in adults: a multicentric clinical study. ORL J Otorhinolaryngol Relat Spec. (1997) 59:23–35. doi: 10.1159/000276901, PMID: 9104746

[13] Lenarz T Kuzma J Weber BP Reuter G Neuburger J Battmer RD. New clarion electrode with positioner: insertion studies. Ann Otol Rhinol Laryngol Suppl. (2000) 185:16–8. doi: 10.1177/0003489400109S1206, PMID: 11140987

[14] HJ REPORT. Audiology seeks common ground. Hear J. (2002) 55:7–8. doi: 10.1097/01.HJ.0000286485.43703.bb

[15] Tykocinski M Saunders E Cohen LT Treaba C Briggs RJ Gibson P. The contour electrode array: safety study and initial patient trials of a new perimodiolar design. Otol Neurotol. (2001) 22:33–41. doi: 10.1097/00129492-200101000-00007, PMID: 11314713

[16] Wright CG Roland PS Kuzma J. Advanced bionics thin lateral and Helix II electrodes: a temporal bone study. Laryngoscope. (2005) 115:2041–5. doi: 10.1097/01.MLG.0000181461.63392.49, PMID: 16319621

[17] van der Jagt MA Briaire JJ Verbist BM Frijns JH. Comparison of the HiFocus Mid-Scala and HiFocus 1J electrode array: angular insertion depths and speech perception outcomes. Audiol Neurootol. (2016) 21:316–25. doi: 10.1159/000448581, PMID: 27871074

[18] Baumgartner WD Jappel A Morera C Gstöttner W Müller J Kiefer J. Outcomes in adults implanted with the FLEXsoft electrode. Acta Otolaryngol. (2007) 127:579–86. doi: 10.1080/00016480600987784 17503226

[19] Klenzner T Richter B Nagursky H Schipper J Laszig R Aschendorff A. Evaluation des Insertionstraumas des nucleus contour advance-Elektrodenträgers im humanen Felsenbeinmodell [Evaluation of the insertion-trauma of the nucleus contour advance electrode-array in a human temporal bone model]. Laryngorhinootologie. (2004) 83:840–4. doi: 10.1055/s-2004-826067, (in German) 15611904

[20] Dhanasingh A. The rationale for FLEX (cochlear implant) electrode with varying array lengths. World J Otorhinolaryngol Head Neck Surg. (2020) 7:45–53. doi: 10.1016/j.wjorl.2019.12.003 33474544 PMC7801259

[21] Driscoll CL Carlson ML Fama AF Lane JI. Evaluation of the hybrid-L24 electrode using microcomputed tomography. Laryngoscope. (2011) 121:1508–16. doi: 10.1002/lary.21837 21541948

[22] Skarzynski H Lorens A Matusiak M Porowski M Skarzynski PH James CJ. Cochlear implantation with the nucleus slim straight electrode in subjects with residual low-frequency hearing. Ear Hear. (2014) 35:e33–43. doi: 10.1097/01.aud.0000444781.15858.f1, PMID: 24556970

[23] Carvalho GM Guimarães AC Danieli F Onuki LC Paschoal JR Bianchini WA. Evaluation of the Digisonic^®^ SP cochlear implant: patient outcomes and fixation system with titanium screws. Braz J Otorhinolaryngol. (2012) 78:56–62. doi: 10.5935/1808-8694.20120034, PMID: 23306569 PMC9446367

[24] Zeng FG Rebscher SJ Fu QJ Chen H Sun X Yin L. Development and evaluation of the Nurotron 26-electrode cochlear implant system. Hear Res. (2015) 322:188–99. doi: 10.1016/j.heares.2014.09.013, PMID: 25281795

[25] Boyle PJ. The rational for a Mid-Scala electrode array. Eur Ann Otorhinolaryngol Head Neck Dis. (2016) 133:S61–2. doi: 10.1016/j.anorl.2016.05.002 27246747

[26] Briggs RJ Tykocinski M Lazsig R Aschendorff A Lenarz T Stöver T. Development and evaluation of the modiolar research array—multi-centre collaborative study in human temporal bones. Cochlear Implants Int. (2011) 12:129–39. doi: 10.1179/1754762811Y0000000007, PMID: 21917200 PMC3159433

[27] Downing M. Electrode designs for protection of the delicate cochlear structures. J Int Adv Otol. (2018) 14:401–3. doi: 10.5152/iao.2018.6461, PMID: 30644381 PMC6354533

[28] Colburn Wade. Webinar: understanding the design and rationale of cochlear nucleus electrode portfolio. Available at: https://pronews.cochlearamericas.com/webinar-nucleus-electrode-portfolio

[29] MED-EL. MED-EL cochlear implant electrode arrays. Available at: https://www.medel.pro/products/electrode-arrays

[30] Van de Heyning P Roland P Lassaletta L Agrawal S Atlas M Baumgartner WD. Suitable electrode choice for robotic-assisted cochlear implant surgery: a systematic literature review of manual electrode insertion adverse events. Front Surg. (2022) 9:823219. doi: 10.3389/fsurg.2022.823219, PMID: 35402479 PMC8987358

[31] Sennaroğlu L Bajin MD. Classification and current management of inner ear malformations. Balkan Med J. (2017) 34:397–411. doi: 10.4274/balkanmedj.2017.0367, PMID: 28840850 PMC5635626

[32] Sun B Dai P Zhou C. Study on 2,747 cases of inner ear malformation for its classification in patient with sensorineural hearing loss. Lin Chung Er Bi Yan Hou Tou Jing Wai Ke Za Zhi. (2015) 29:45–7. PMID: (in Chinese) 25966554

[33] Sugarova S Kuzovkov V Altamimi F Vetrichelvan J Prasad R Kedves A. Applications of visualizing cochlear basal turn in cochlear implantation. Laryngoscope Investig Otolaryngol. (2023) 8:1666–72. doi: 10.1002/lio2.1187, PMID: 38130266 PMC10731499

[34] Graham JM Phelps PD Michaels L. Congenital malformations of the ear and cochlear implantation in children: review and temporal bone report of common cavity. J Laryngol Otol Suppl. (2000) 25:1–14. doi: 10.1258/0022215001904842, PMID: 10824232

[35] Kim BJ Jeon H Kim Y Lee SY Han JH Yi N. Long-term audiologic outcomes and potential outcome predictors of cochlear implantation in cochlear aplasia with dilated vestibule: a case series. Clin Otolaryngol. (2022) 47:599–605. doi: 10.1111/coa.13952, PMID: 35653220

[36] Alexiades G Dhanasingh A Jolly C. Method to estimate the complete and two-turn cochlear duct length. Otol Neurotol. (2015) 36:904–7. doi: 10.1097/MAO.0000000000000620, PMID: 25299827

[37] Pearl MS Roy A Limb CJ. High-resolution secondary reconstructions with the use of flat panel CT in the clinical assessment of patients with cochlear implants. AJNR Am J Neuroradiol. (2014) 35:1202–8. doi: 10.3174/ajnr.A3814, PMID: 24371026 PMC7965126

[38] Khurayzi T Dhanasingh A Almuhawas F Alsanosi A. Shape of the cochlear basal turn: an indicator for an optimal *electrode-to-modiolus* proximity with precurved electrode type. Ear Nose Throat J. (2021) 100:38–43. doi: 10.1177/0145561320920965, PMID: 32330070

[39] Rask-Andersen H Erixon E Kinnefors A Löwenheim H Schrott-Fischer A Liu W. Anatomy of the human cochlea—implications for cochlear implantation. Cochlear Implants Int. (2011) 12:S13–S8. doi: 10.1179/146701011X13001035752174 21756464

[40] Todt I Basta D Eisenschenk A Ernst A. The “pull-back” technique for nucleus 24 perimodiolar electrode insertion. Otolaryngol Head Neck Surg. (2005) 132:751–4. doi: 10.1016/j.otohns.2005.01.046, PMID: 15886630

[41] Dhanasingh AE Rajan G van de Heyning P. Presence of the spiral ganglion cell bodies beyond the basal turn of the human cochlea. Cochlear Implants Int. (2020) 21:145–52. doi: 10.1080/14670100.2019.1694226, PMID: 31771498

[42] Li H Helpard L Ekeroot J Rohani SA Zhu N Rask-Andersen H. Three-dimensional tonotopic mapping of the human cochlea based on synchrotron radiation phase-contrast imaging. Sci Rep. (2021) 11:4437. doi: 10.1038/s41598-021-83225-w, PMID: 33627724 PMC7904830

[43] Starovoyt A Pyka G Putzeys T Balcaen T Wouters J Kerckhofs G. Human cochlear microstructures at risk of electrode insertion trauma, elucidated in 3D with contrast-enhanced microCT. Sci Rep. (2023) 13:2191. doi: 10.1038/s41598-023-29401-6, PMID: 36750646 PMC9905077

[44] Eshraghi AA Yang NW Balkany TJ. Comparative study of cochlear damage with three perimodiolar electrode designs. Laryngoscope. (2003) 113:415–9. doi: 10.1097/00005537-200303000-00005, PMID: 12616189

[45] Jwair S Prins A Wegner I Stokroos RJ Versnel H Thomeer HGXM. Scalar translocation comparison between lateral wall and perimodiolar cochlear implant arrays—a meta-analysis. Laryngoscope. (2021) 131:1358–68. doi: 10.1002/lary.29224, PMID: 33159469 PMC8246990

[46] Shaul C Dragovic AS Stringer AK O’Leary SJ Briggs RJ. Scalar localisation of peri-modiolar electrodes and speech perception outcomes. J Laryngol Otol. (2018) 132:1000–6. doi: 10.1017/S0022215118001871, PMID: 30370884

[47] Ishiyama A Risi F Boyd P. Potential insertion complications with cochlear implant electrodes. Cochlear Implants Int. (2020) 21:206–19. doi: 10.1080/14670100.2020.1730066 32079506

[48] Goh X Harvey L Axon PR Donnelly NP Tysome JR Borsetto D. Frequency of electrode migration after cochlear implantation in the early postoperative period. What are associated risk factors? Clin Otolaryngol. (2023) 48:638–47. doi: 10.1111/coa.14062, PMID: 37051731

[49] Nordfalk KF Rasmussen K Hopp E Bunne M Silvola JT Jablonski GE. Insertion depth in cochlear implantation and outcome in residual hearing and vestibular function. Ear Hear. (2016) 37:e129–37. doi: 10.1097/AUD.0000000000000241, PMID: 26524566

[50] Canfarotta MW Dillon MT Brown KD Pillsbury HC Dedmon MM O’Connell BP. Incidence of complete insertion in cochlear implant recipients of long lateral wall arrays. Otolaryngol Head Neck Surg. (2021) 165:571–7. doi: 10.1177/0194599820987456, PMID: 33588627 PMC8943918

[51] Lee J Nadol JB Jr Eddington DK. Factors associated with incomplete insertion of electrodes in cochlear implant surgery: a histopathologic study. Audiol Neurootol. (2011) 16:69–81. doi: 10.1159/000316445, PMID: 20571258 PMC2948664

[52] Torres R Drouillard M De Seta D Bensimon JL Ferrary E Sterkers O. Cochlear implant insertion axis into the basal turn: a critical factor in electrode array translocation. Otol Neurotol. (2018) 39:168–76. doi: 10.1097/MAO.0000000000001648, PMID: 29194215

[53] Berg KA Noble JH Dawant BM Dwyer RT Labadie RF Gifford RH. Speech recognition with cochlear implants as a function of the number of channels: effects of electrode placement. J Acoust Soc Am. (2020) 147:3646–56. doi: 10.1121/10.0001316, PMID: 32486813 PMC7255811

[54] Jeong J Kim M Heo JH Bang MY Bae MR Kim J. Intraindividual comparison of psychophysical parameters between perimodiolar and lateral-type electrode arrays in patients with bilateral cochlear implants. Otol Neurotol. (2015) 36:228–34. doi: 10.1097/MAO.0000000000000672, PMID: 25473955

[55] Fitzgerald MB Shapiro WH McDonald PD Neuburger HS Ashburn-Reed S Immerman S. The effect of perimodiolar placement on speech perception and frequency discrimination by cochlear implant users. Acta Otolaryngol. (2007) 127:378–83. doi: 10.1080/00016480701258671, PMID: 17453457

[56] Doshi J Johnson P Mawman D Green K Bruce IA Freeman S. Straight versus modiolar hugging electrodes: does one perform better than the other? Otol Neurotol. (2015) 36:223–7. doi: 10.1097/MAO.0000000000000603, PMID: 25415467

[57] Garaycochea O Manrique-Huarte R Lazaro C Huarte A Prieto C Alvarez de Linera-Alperi M. Comparative study of two different perimodiolar and a straight cochlear implant electrode array: surgical and audiological outcomes. Eur Arch Otorrinolaringol. (2020) 277:69–76. doi: 10.1007/s00405-019-05680-6, PMID: 31637478

[58] Sturm JJ Patel V Dibelius G Kuhlmey M Kim AH. Comparative performance of lateral wall and perimodiolar cochlear implant arrays. Otol Neurotol. (2021) 42:532–9. doi: 10.1097/MAO.0000000000002997 33710993

[59] MacPhail ME Connell NT Totten DJ Gray MT Pisoni D Yates CW. Speech recognition outcomes in adults with slim straight and slim modiolar cochlear implant electrode arrays. Otolaryngol Head Neck Surg. (2022) 166:943–50. doi: 10.1177/01945998211036339, PMID: 34399646

[60] Heutink F Verbist BM van der Woude WJ Meulman TJ Briaire JJ Frijns JHM. Factors influencing speech perception in adults with a cochlear implant. Ear Hear. (2021) 42:949–60. doi: 10.1097/AUD.0000000000000988, PMID: 33480623 PMC8221708

[61] Holder JT Yawn RJ Nassiri AM Dwyer RT Rivas A Labadie RF. Matched cohort comparison indicates superiority of precurved electrode arrays. Otol Neurotol. (2019) 40:1160–6. doi: 10.1097/MAO.0000000000002366, PMID: 31469799 PMC6999087

[62] Patro A Lindquist NR Schauwecker N Holder JT Perkins EL Haynes DS. Comparison of speech recognition and hearing preservation outcomes between the Mid-Scala and lateral wall electrode arrays. Otol Neurotol. (2023) 45:52–7. doi: 10.1097/MAO.0000000000004064 38013487 PMC10842140

[63] Smullen JL Polak M Hodges AV Payne SB King JE 3rd Telischi FF. Facial nerve stimulation after cochlear implantation. Laryngoscope. (2005) 115:977–82. doi: 10.1097/01.MLG.0000163100.37713.C6 15933504

[64] Berrettini S Vito de A Bruschini L Passetti S Forli F. Facial nerve stimulation after cochlear implantation: our experience. Acta Otorhinolaryngol Ital. (2011) 31:11–6. PMID: 21808458 PMC3146332

[65] Seyyedi M Herrmann BS Eddington DK Nadol JB Jr. Jwair. Otol Neurotol. (2013) 34:1603–9. doi: 10.1097/MAO.0b013e3182979398, PMID: 23928519 PMC3825753

[66] Van Horn A Hayden C Mahairas AD Leader P Bush ML. Factors influencing aberrant facial nerve stimulation following cochlear implantation: a systematic review and meta-analysis. Otol Neurotol. (2020) 41:1050–9. doi: 10.1097/MAO.0000000000002693, PMID: 32558747

[67] Alahmadi A Abdelsamad Y Yousef M Alhabib SF Alshalan A Hamed N. Risk factors and management strategies of inadvertent facial nerve stimulation in cochlear implant recipients: a systematic review. Laryngoscope Investig Otolaryngol. (2023) 8:1345–56. doi: 10.1002/lio2.1121, PMID: 37899846 PMC10601549

[68] Bahmer A Adel Y Baumann U. Preventing facial nerve stimulation by triphasic pulse stimulation in cochlear implant users: intraoperative recordings. Otol Neurotol. (2017) 38:e438–44. doi: 10.1097/MAO.0000000000001603, PMID: 29065088

[69] Eitutis ST Carlyon RP Tam YC Salorio-Corbetto M Vanat Z Tebbutt K. Management of severe facial nerve cross stimulation by cochlear implant replacement to change pulse shape and grounding configuration: a case-series. Otol Neurotol. (2022) 43:452–9. doi: 10.1097/MAO.0000000000003493, PMID: 35085112 PMC8915992

[70] Hoffmann JAC Warnecke A Timm ME Kludt E Prenzler NK Gärtner L. Cochlear implantation in obliterated cochlea: a retrospective analysis and comparison between the IES stiff custom-made device and the split-array and regular electrodes. J Clin Med. (2022) 11:6090. doi: 10.3390/jcm11206090, PMID: 36294411 PMC9605638

[71] Danielian A Ishiyama G Lopez IA Ishiyama A. Predictors of fibrotic and bone tissue formation with 3-D reconstructions of post-implantation human temporal bones. Otol Neurotol. (2021) 42:e942–8. doi: 10.1097/MAO.0000000000003106, PMID: 33710156 PMC8282738

[72] Jwair S Versnel H Stokroos RJ Thomeer HGXM. The effect of the surgical approach and cochlear implant electrode on the structural integrity of the cochlea in human temporal bones. Sci Rep. (2022) 12:17068. doi: 10.1038/s41598-022-21399-7, PMID: 36224234 PMC9556579

[73] Geerardyn A Zhu M Klabbers T Huinck W Mylanus E Nadol JB Jr. Human histology after structure preservation Cochlear implantation via round window insertion. Laryngoscope. (2023) 134:945–53. doi: 10.1002/lary.30900 37493203

[74] Ketterer MC Brückerhoff K Arndt S Beck R Aschendorff A. Insertion of a second electrode array-a rare complication of CI reimplantation. HNO. (2023) 72:63–5. doi: 10.1007/s00106-023-01364-0, (in English) 37943372 PMC10798908

