# Supplementary material for: Cochlear implant electrode design for safe and effective treatment
Source: Front Neurol. 2024 May 2;15:1348439. doi: 10.3389/fneur.2024.1348439 (PMC11096578; doi:10.3389/fneur.2024.1348439)
Supplement: Supplementary file 1 [file Table_1.DOCX]

Supplement 1.

1. Canfarotta MW, Dillon MT, Brown KD, Pillsbury HC, Dedmon MM, O'Connell BP. Insertion Depth and Cochlear Implant Speech Recognition Outcomes: A Comparative Study of 28- and 31.5-mm Lateral Wall Arrays. Otol Neurotol. 2022 Feb 1;43(2):183-189. doi: 10.1097/MAO.0000000000003416. PMID: 34772886; PMCID: PMC8752482.
2. Thimsen V, Mantsopoulos K, Liebscher T, Taha L, Eisenhut F, Iro H, Hoppe U, Hornung J. Association between lateral wall electrode array insertion parameters and audiological outcomes in bilateral cochlear implantation. Eur Arch Otorhinolaryngol. 2023 Jun;280(6):2707-2714. doi: 10.1007/s00405-022-07756-2. Epub 2022 Nov 27. PMID: 36436080; PMCID: PMC10175364.
3. Razmovski T, Bester C, Collins A, Tan E, O'Leary SJ. Four-Point Impedance Changes After Cochlear Implantation for Lateral Wall and Perimodiolar Implants. Otol Neurotol. 2022 Dec 1;43(10):e1107-e1114. doi: 10.1097/MAO.0000000000003732. PMID: 36351225.
4. Fan T, Xiang MY, Li Y, Gong JM, Wu T, Wang Y, Xu J, Wang YF, Li J. Effect of Electrode Insertion Angle on Cochlear Implantation Outcomes in Adult and Children Patients with Sensorineural Hearing Loss. Oxid Med Cell Longev. 2022 Aug 23;2022:9914716. doi: 10.1155/2022/9914716. PMID: 36052159; PMCID: PMC9427248.
5. Andersen SAW, Keith JP, Hittle B, Riggs WJ, Adunka O, Wiet GJ, Powell KA. Automated Calculation of Cochlear Implant Electrode Insertion Parameters in Clinical Cone-Beam CT. Otol Neurotol. 2022 Feb 1;43(2):199-205. doi: 10.1097/MAO.0000000000003432. PMID: 34789695.
6. Högerle C, Englhard A, Simon F, Grüninger I, Mlynski R, Hempel JM, Müller J. Cochlear Implant Electrode Tip Fold-Over: Our Experience With Long and Flexible Electrode. Otol Neurotol. 2022 Jan 1;43(1):64-71. doi: 10.1097/MAO.0000000000003362. PMID: 34619728.
7. Lee SY, Kim YS, Jo HD, Kim Y, Carandang M, Huh G, Choi BY. Effects of in vivo repositioning of slim modiolar electrodes on electrical thresholds and speech perception. Sci Rep. 2021 Jul 23;11(1):15135. doi: 10.1038/s41598-021-94668-6. PMID: 34302030; PMCID: PMC8302625.
8. Spiegel JL, Polterauer D, Hempel JM, Canis M, Spiro JE, Müller J. Variation of the cochlear anatomy and cochlea duct length: analysis with a new tablet-based software. Eur Arch Otorhinolaryngol. 2022 Apr;279(4):1851-1861. doi: 10.1007/s00405-021-06889-0. Epub 2021 May 29. PMID: 34050805; PMCID: PMC8930796.
9. Goehring T, Archer-Boyd AW, Arenberg JG, Carlyon RP. The effect of increased channel interaction on speech perception with cochlear implants. Sci Rep. 2021 May 17;11(1):10383. doi: 10.1038/s41598-021-89932-8. PMID: 34001987; PMCID: PMC8128897.
10. Labadie RF, Riojas K, Von Wahlde K, Mitchell J, Bruns T, Webster R 3rd, Dawant B, Fitzpatrick JM, Noble J. Clinical Implementation of Second-generation Minimally Invasive Image-guided Cochlear Implantation Surgery. Otol Neurotol. 2021 Jun 1;42(5):702-705. doi: 10.1097/MAO.0000000000003025. PMID: 33967246.
11. Morrel WG, Manzoor NF, Dawant BM, Noble JH, Labadie RF. Intraoperative Correction of Cochlear Implant Electrode Translocation. Audiol Neurootol. 2021 Apr 29:1-5. doi: 10.1159/000515684. Epub ahead of print. PMID: 33915536.
12. Canfarotta MW, Dillon MT, Brown KD, Pillsbury HC, Dedmon MM, O'Connell BP. Incidence of Complete Insertion in Cochlear Implant Recipients of Long Lateral Wall Arrays. Otolaryngol Head Neck Surg. 2021 Oct;165(4):571-577. doi: 10.1177/0194599820987456. Epub 2021 Feb 16. PMID: 33588627.
13. Heutink F, Verbist BM, van der Woude WJ, Meulman TJ, Briaire JJ, Frijns JHM, Vart P, Mylanus EAM, Huinck WJ. Factors Influencing Speech Perception in Adults With a Cochlear Implant. Ear Hear. 2021 Jul-Aug 01;42(4):949-960. doi: 10.1097/AUD.0000000000000988. PMID: 33480623; PMCID: PMC8221708.
14. Canfarotta MW, O'Connell BP, Giardina CK, Buss E, Brown KD, Dillon MT, Rooth MA, Pillsbury HC, Buchman CA, Adunka OF, Fitzpatrick DC. Relationship Between Electrocochleography, Angular Insertion Depth, and Cochlear Implant Speech Perception Outcomes. Ear Hear. 2021 Jul-Aug 01;42(4):941-948. doi: 10.1097/AUD.0000000000000985. PMID: 33369942; PMCID: PMC8217403.
15. Canfarotta MW, O'Connell BP, Giardina CK, Buss E, Brown KD, Dillon MT, Rooth MA, Pillsbury HC, Buchman CA, Adunka OF, Fitzpatrick DC. Relationship Between Electrocochleography, Angular Insertion Depth, and Cochlear Implant Speech Perception Outcomes. Ear Hear. 2021 July/Aug;42(4):941-948. doi: 10.1097/AUD.0000000000000985. PMID: 33369942; PMCID: PMC8217403.
16. Lenarz T, Buechner A, Lesinski-Schiedat A, Timm M, Salcher R. Hearing Preservation With a New Atraumatic Lateral Wall Electrode. Otol Neurotol. 2020 Sep;41(8):e993-e1003. doi: 10.1097/MAO.0000000000002714. PMID: 32569147.
17. Khan MMR, Labadie RF, Noble JH. Preoperative prediction of angular insertion depth of lateral wall cochlear implant electrode arrays. J Med Imaging (Bellingham). 2020 May;7(3):031504. doi: 10.1117/1.JMI.7.3.031504. Epub 2020 Jun 3. PMID: 32509912; PMCID: PMC7269369.
18. Canfarotta MW, Dillon MT, Buchman CA, Buss E, O'Connell BP, Rooth MA, King ER, Pillsbury HC, Adunka OF, Brown KD. Long-Term Influence of Electrode Array Length on Speech Recognition in Cochlear Implant Users. Laryngoscope. 2021 Apr;131(4):892-897. doi: 10.1002/lary.28949. Epub 2020 Aug 1. PMID: 32738069; PMCID: PMC7855603.
19. Noble AR, Christianson E, Norton SJ, Ou HC, Phillips GS, Khalatbari H, Friedman SD, Horn DL. Reliability of Measuring Insertion Depth in Cochlear Implanted Infants and Children Using Cochlear View Radiography. Otolaryngol Head Neck Surg. 2020 Oct;163(4):822-828. doi: 10.1177/0194599820921857. Epub 2020 May 26. PMID: 32450736.
20. Canfarotta MW, Dillon MT, Buss E, Pillsbury HC, Brown KD, O'Connell BP. Frequency-to-Place Mismatch: Characterizing Variability and the Influence on Speech Perception Outcomes in Cochlear Implant Recipients. Ear Hear. 2020 Sep/Oct;41(5):1349-1361. doi: 10.1097/AUD.0000000000000864. PMID: 32205726; PMCID: PMC8407755.
21. Canfarotta MW, O'Connell BP, Buss E, Pillsbury HC, Brown KD, Dillon MT. Influence of Age at Cochlear Implantation and Frequency-to-Place Mismatch on Early Speech Recognition in Adults. Otolaryngol Head Neck Surg. 2020 Jun;162(6):926-932. doi: 10.1177/0194599820911707. Epub 2020 Mar 17. PMID: 32178574.
22. Nassiri AM, Yawn RJ, Holder JT, Dwyer RT, O'Malley MR, Bennett ML, Labadie RF, Rivas A. Hearing Preservation Outcomes Using a Precurved Electrode Array Inserted With an External Sheath. Otol Neurotol. 2020 Jan;41(1):33-38. doi: 10.1097/MAO.0000000000002426. PMID: 31746820; PMCID: PMC6910978.
23. Rivas A, Yawn RJ, Kim AH, Driscoll C, Cullen R, Rebscher SJ, Isaacson B. A New Lateral Wall Electrode: Evaluation of Surgical Handling, Radiographic Placement, and Histological Appraisal of Insertion Trauma. Otol Neurotol. 2019 Jun;40(5S Suppl 1):S23-S28. doi: 10.1097/MAO.0000000000002210. PMID: 31225819.
24. Rathgeb C, Demattè M, Yacoub A, Anschuetz L, Wagner F, Mantokoudis G, Caversaccio M, Wimmer W. Clinical Applicability of a Preoperative Angular Insertion Depth Prediction Method for Cochlear Implantation. Otol Neurotol. 2019 Sep;40(8):1011-1017. doi: 10.1097/MAO.0000000000002304. PMID: 31419213.
25. Canfarotta MW, Dillon MT, Buss E, Pillsbury HC, Brown KD, O'Connell BP. Validating a New Tablet-based Tool in the Determination of Cochlear Implant Angular Insertion Depth. Otol Neurotol. 2019 Sep;40(8):1006-1010. doi: 10.1097/MAO.0000000000002296. PMID: 31290802; PMCID: PMC6697191.
26. Abd El Aziz TT, El Fiky L, Shalaby MH, Essam A. Radiological evaluation of inner ear trauma after cochlear implant surgery by cone beam CT(CBCT). Eur Arch Otorhinolaryngol. 2019 Oct;276(10):2697-2703. doi: 10.1007/s00405-019-05507-4. Epub 2019 Jun 13. PMID: 31214825.
27. Yamamoto N, Okano T, Yamazaki H, Hiraumi H, Sakamoto T, Ito J, Omori K. Intraoperative Evaluation of Cochlear Implant Electrodes Using Mobile Cone-Beam Computed Tomography. Otol Neurotol. 2019 Feb;40(2):177-183. doi: 10.1097/MAO.0000000000002097. PMID: 30624399.
28. An SY, An CH, Lee KY, Jang JH, Choung YH, Lee SH. Diagnostic role of cone beam computed tomography for the position of straight array. Acta Otolaryngol. 2018 Apr;138(4):375-381. doi: 10.1080/00016489.2017.1404639. Epub 2017 Nov 26. Erratum in: Acta Otolaryngol. 2018 Apr;138(4): i. PMID: 29172857.
29. Skarzynski H, Matusiak M, Furmanek M, Pilka A, Wlodarczyk E, Oldak M, Skarzynski PH. Radiologic measurement of cochlea and hearing preservation rate using slim straight electrode (CI422) and round window approach. Acta Otorhinolaryngol Ital. 2018 Oct;38(5):468-475. doi: 10.14639/0392-100X-1579. PMID: 30498276; PMCID: PMC6265667.
30. Dietz A, Gazibegovic D, Tervaniemi J, Vartiainen VM, Löppönen H. Insertion characteristics and placement of the Mid-Scala electrode array in human temporal bones using detailed cone beam computed tomography. Eur Arch Otorhinolaryngol. 2016 Dec;273(12):4135-4143. doi: 10.1007/s00405-016-4099-x. Epub 2016 May 19. PMID: 27194346.
31. Iso-Mustajärvi M, Matikka H, Risi F, Sipari S, Koski T, Willberg T, Lehtimäki A, Tervaniemi J, Löppönen H, Dietz A. A New Slim Modiolar Electrode Array for Cochlear Implantation: A Radiological and Histological Study. Otol Neurotol. 2017 Oct;38(9): e327-e334. doi: 10.1097/MAO.0000000000001542. PMID: 28796083.
32. O'Connell BP, Hunter JB, Haynes DS, Holder JT, Dedmon MM, Noble JH, Dawant BM, Wanna GB. Insertion depth impacts speech perception and hearing preservation for lateral wall electrodes. Laryngoscope. 2017 Oct;127(10):2352-2357. doi: 10.1002/lary.26467. Epub 2017 Mar 17. PMID: 28304096; PMCID: PMC5825186.
33. van der Jagt MA, Briaire JJ, Verbist BM, Frijns JH. Comparison of the HiFocus Mid-Scala and HiFocus 1J Electrode Array: Angular Insertion Depths and Speech Perception Outcomes. Audiol Neurootol. 2016;21(5):316-325. doi: 10.1159/000448581. Epub 2016 Nov 21. PMID: 27871074.
34. Roy AT, Penninger RT, Pearl MS, Wuerfel W, Jiradejvong P, Carver C, Buechner A, Limb CJ. Deeper Cochlear Implant Electrode Insertion Angle Improves Detection of Musical Sound Quality Deterioration Related to Bass Frequency Removal. Otol Neurotol. 2016 Feb;37(2):146-51. doi: 10.1097/MAO.0000000000000932. PMID: 26669557.
35. Svrakic M, Roland JT Jr, McMenomey SO, Svirsky MA. Initial Operative Experience and Short-term Hearing Preservation Results With a Mid-scala Cochlear Implant Electrode Array. Otol Neurotol. 2016 Dec;37(10):1549-1554. doi: 10.1097/MAO.0000000000001238. PMID: 27755356; PMCID: PMC5104204.
36. Benghalem A, Gazibegovic D, Saadi F, Tazi-Chaoui Z. Use of a mid-scala and a lateral wall electrode in children: insertion depth and hearing preservation. Acta Otolaryngol. 2017 Jan;137(1):1-7. doi: 10.1080/00016489.2016.1208367. Epub 2016 Jul 29. PMID: 27472299.
37. O'Connell BP, Cakir A, Hunter JB, Francis DO, Noble JH, Labadie RF, Zuniga G, Dawant BM, Rivas A, Wanna GB. Electrode Location and Angular Insertion Depth Are Predictors of Audiologic Outcomes in Cochlear Implantation. Otol Neurotol. 2016 Sep;37(8):1016-23. doi: 10.1097/MAO.0000000000001125. PMID: 27348391; PMCID: PMC4983244.
38. Nordfalk KF, Rasmussen K, Hopp E, Bunne M, Silvola JT, Jablonski GE. Insertion Depth in Cochlear Implantation and Outcome in Residual Hearing and Vestibular Function. Ear Hear. 2016 Mar-Apr;37(2):e129-37. doi: 10.1097/AUD.0000000000000241. PMID: 26524566.
39. Frisch CD, Carlson ML, Lane JI, Driscoll CL. Evaluation of a new mid-scala cochlear implant electrode using microcomputed tomography. Laryngoscope. 2015 Dec;125(12):2778-83. doi: 10.1002/lary.25347. Epub 2015 May 6. PMID: 25946683.
40. Skarżyński H, Matusiak M, Furmanek M, Skarzyński PH. Deep insertion - round window approach by using SRA electrode. Cochlear Implants Int. 2014 May;15 Suppl 1:S4-7. doi: 10.1179/1467010014Z.000000000159. PMID: 24869441.
41. Schatzer R, Vermeire K, Visser D, Krenmayr A, Kals M, Voormolen M, Van de Heyning P, Zierhofer C. Electric-acoustic pitch comparisons in single-sided-deaf cochlear implant users: frequency-place functions and rate pitch. Hear Res. 2014 Mar;309: 26-35. doi: 10.1016/j.heares.2013.11.003. Epub 2013 Nov 16. PMID: 24252455.
42. Pearl MS, Roy A, Limb CJ. High-resolution secondary reconstructions with the use of flat panel CT in the clinical assessment of patients with cochlear implants. AJNR Am J Neuroradiol. 2014 Jun;35(6):1202-8. doi: 10.3174/ajnr.A3814. Epub 2013 Dec 26. PMID: 24371026; PMCID: PMC7965126.
43. Trieger A, Schulze A, Schneider M, Zahnert T, Mürbe D. In vivo measurements of the insertion depth of cochlear implant arrays using flat-panel volume computed tomography. Otol Neurotol. 2011 Jan;32(1):152-7. doi: 10.1097/MAO.0b013e3181fcf04d. PMID: 20962701.
44. Radeloff A, Mack M, Baghi M, Gstoettner WK, Adunka OF. Variance of angular insertion depths in free-fitting and perimodiolar cochlear implant electrodes. Otol Neurotol. 2008 Feb;29(2):131-6. doi: 10.1097/MAO.0b013e318157f0ea. PMID: 18090204.
45. Xu J, Xu SA, Cohen LT, Clark GM. Cochlear view: postoperative radiography for cochlear implantation. Am J Otol. 2000 Jan;21(1):49-56. PMID: 10651435.
