# Supplementary material for: Cochlear implant electrode design for safe and effective treatment
Source: Front Neurol. 2024 May 2;15:1348439. doi: 10.3389/fneur.2024.1348439 (PMC11096578; doi:10.3389/fneur.2024.1348439)
Supplement: Supplementary file 2 [file Table_2.DOCX]

**Supplement 2.**

1. Alothman N, Almuhawas F, Badghaish R, Alotaibi AH, Alhabib SF, Alzhrani F, Hagr A. Cochlear Implantation in Pediatrics: The Effect of Cochlear Coverage. J Pers Med. 2023 Mar 21;13(3):562. doi: 10.3390/jpm13030562. PMID: 36983743; PMCID: PMC10051355.
2. Canfarotta MW, Dillon MT, Brown KD, Pillsbury HC, Dedmon MM, O'Connell BP. Insertion Depth and Cochlear Implant Speech Recognition Outcomes: A Comparative Study of 28- and 31.5-mm Lateral Wall Arrays. Otol Neurotol. 2022 Feb 1;43(2):183-189. doi: 10.1097/MAO.0000000000003416. PMID: 34772886; PMCID: PMC8752482.
3. Fan T, Xiang MY, Li Y, Gong JM, Wu T, Wang Y, Xu J, Wang YF, Li J. Effect of Electrode Insertion Angle on Cochlear Implantation Outcomes in Adult and Children Patients with Sensorineural Hearing Loss. Oxid Med Cell Longev. 2022 Aug 23;2022:9914716. doi: 10.1155/2022/9914716. PMID: 36052159; PMCID: PMC9427248.
4. Lo Russo F, Conte G, Di Berardino F, Cavicchiolo S, Casale S, Caschera L, Lombardi L, Triulzi F, Zanetti D. Impact of Cochlear Implant Array Placement on Speech Perception. Clin Neuroradiol. 2022 Mar;32(1):175-183. doi: 10.1007/s00062-021-01046-w. Epub 2021 Jun 17. PMID: 34142163.
5. Heutink F, Verbist BM, van der Woude WJ, Meulman TJ, Briaire JJ, Frijns JHM, Vart P, Mylanus EAM, Huinck WJ. Factors Influencing Speech Perception in Adults With a Cochlear Implant. Ear Hear. 2021 July/Aug;42(4):949-960. doi: 10.1097/AUD.0000000000000988. PMID: 33480623; PMCID: PMC8221708.
6. Canfarotta MW, Dillon MT, Buchman CA, Buss E, O'Connell BP, Rooth MA, King ER, Pillsbury HC, Adunka OF, Brown KD. Long-Term Influence of Electrode Array Length on Speech Recognition in Cochlear Implant Users. Laryngoscope. 2021 Apr;131(4):892-897. doi: 10.1002/lary.28949. Epub 2020 Aug 1. PMID: 32738069; PMCID: PMC7855603.
7. Canfarotta MW, Dillon MT, Buss E, Pillsbury HC, Brown KD, O'Connell BP. Frequency-to-Place Mismatch: Characterizing Variability and the Influence on Speech Perception Outcomes in Cochlear Implant Recipients. Ear Hear. 2020 Sep/Oct;41(5):1349-1361. doi: 10.1097/AUD.0000000000000864. PMID: 32205726; PMCID: PMC8407755.
8. Nassiri AM, Yawn RJ, Holder JT, Dwyer RT, O'Malley MR, Bennett ML, Labadie RF, Rivas A. Hearing Preservation Outcomes Using a Precurved Electrode Array Inserted With an External Sheath. Otol Neurotol. 2020 Jan;41(1):33-38. doi: 10.1097/MAO.0000000000002426. PMID: 31746820; PMCID: PMC6910978.
9. Selleck AM, Park LR, Brown KD. Factors Influencing Pediatric Cochlear Implant Outcomes: Carolina Sibling Study. Otol Neurotol. 2019 Oct;40(9):1148-1152. doi: 10.1097/MAO.0000000000002342. PMID: 31436630.
10. Chakravorti S, Noble JH, Gifford RH, Dawant BM, O'Connell BP, Wang J, Labadie RF. Further Evidence of the Relationship Between Cochlear Implant Electrode Positioning and Hearing Outcomes. Otol Neurotol. 2019 Jun;40(5):617-624. doi: 10.1097/MAO.0000000000002204. PMID: 31083083; PMCID: PMC6788798.
11. O'Connell BP, Hunter JB, Haynes DS, Holder JT, Dedmon MM, Noble JH, Dawant BM, Wanna GB. Insertion depth impacts speech perception and hearing preservation for lateral wall electrodes. Laryngoscope. 2017 Oct;127(10):2352-2357. doi: 10.1002/lary.26467. Epub 2017 Mar 17. PMID: 28304096; PMCID: PMC5825186.
12. Hilly O, Smith L, Hwang E, Shipp D, Symons S, Nedzelski JM, Chen JM, Lin VY. Depth of Cochlear Implant Array Within the Cochlea and Performance Outcome. Ann Otol Rhinol Laryngol. 2016 Nov;125(11):886-892. doi: 10.1177/0003489416660111. Epub 2016 Jul 21. PMID: 27443343.
13. De Seta D, Nguyen Y, Bonnard D, Ferrary E, Godey B, Bakhos D, Mondain M, Deguine O, Sterkers O, Bernardeschi D, Mosnier I. The Role of Electrode Placement in Bilateral Simultaneously Cochlear-Implanted Adult Patients. Otolaryngol Head Neck Surg. 2016 Sep;155(3):485-93. doi: 10.1177/0194599816645774. Epub 2016 May 10. PMID: 27165685.
14. Nayak G, Panda NK, Banumathy N, Munjal S, Khandelwal N, Saxena A. Deeper insertion of electrode array result in better rehabilitation outcomes - Do we have evidence? Int J Pediatr Otorhinolaryngol. 2016 Mar;82:47-53. doi: 10.1016/j.ijporl.2015.12.028. Epub 2016 Jan 7. PMID: 26857315.
15. Roy AT, Penninger RT, Pearl MS, Wuerfel W, Jiradejvong P, Carver C, Buechner A, Limb CJ. Deeper Cochlear Implant Electrode Insertion Angle Improves Detection of Musical Sound Quality Deterioration Related to Bass Frequency Removal. Otol Neurotol. 2016 Feb;37(2):146-51. doi: 10.1097/MAO.0000000000000932. PMID: 26669557.
16. Roy AT, Penninger RT, Pearl MS, Wuerfel W, Jiradejvong P, Carver C, Buechner A, Limb CJ. Deeper Cochlear Implant Electrode Insertion Angle Improves Detection of Musical Sound Quality Deterioration Related to Bass Frequency Removal. Otol Neurotol. 2016 Feb;37(2):146-51. doi: 10.1097/MAO.0000000000000932. PMID: 26669557.
17. Holden LK, Finley CC, Firszt JB, Holden TA, Brenner C, Potts LG, Gotter BD, Vanderhoof SS, Mispagel K, Heydebrand G, Skinner MW. Factors affecting open-set word recognition in adults with cochlear implants. Ear Hear. 2013 May-Jun;34(3):342-60. doi: 10.1097/AUD.0b013e3182741aa7. PMID: 23348845; PMCID: PMC3636188.
18. Lee J, Nadol JB Jr, Eddington DK. Depth of electrode insertion and postoperative performance in humans with cochlear implants: a histopathologic study. Audiol Neurootol. 2010;15(5):323-31. doi: 10.1159/000289571. Epub 2010 Mar 4. PMID: 20203481; PMCID: PMC2919426.
19. Finley CC, Holden TA, Holden LK, Whiting BR, Chole RA, Neely GJ, Hullar TE, Skinner MW. Role of electrode placement as a contributor to variability in cochlear implant outcomes. Otol Neurotol. 2008 Oct;29(7):920-8. doi: 10.1097/MAO.0b013e318184f492. PMID: 18667935; PMCID: PMC2663852.
20. Khan AM, Handzel O, Burgess BJ, Damian D, Eddington DK, Nadol JB Jr. Is word recognition correlated with the number of surviving spiral ganglion cells and electrode insertion depth in human subjects with cochlear implants? Laryngoscope. 2005 Apr;115(4):672-7. doi: 10.1097/01.mlg.0000161335.62139.80. PMID: 15805879.
21. Yukawa K, Cohen L, Blamey P, Pyman B, Tungvachirakul V, O'Leary S. Effects of insertion depth of cochlear implant electrodes upon speech perception. Audiol Neurootol. 2004 May-Jun;9(3):163-72. doi: 10.1159/000077267. PMID: 15084821.
22. Skinner MW, Ketten DR, Holden LK, Harding GW, Smith PG, Gates GA, Neely JG, Kletzker GR, Brunsden B, Blocker B. CT-derived estimation of cochlear morphology and electrode array position in relation to word recognition in Nucleus-22 recipients. J Assoc Res Otolaryngol. 2002 Sep;3(3):332-50. doi: 10.1007/s101620020013. Epub 2002 Feb 27. PMID: 12382107; PMCID: PMC3202410.
