# Supplementary material for: Cochlear implant electrode design for safe and effective treatment
Source: Front Neurol. 2024 May 2;15:1348439. doi: 10.3389/fneur.2024.1348439 (PMC11096578; doi:10.3389/fneur.2024.1348439)
Supplement: Supplementary file 3 [file Table_3.DOCX]

**Supplement 3**

1. Lyutenski S, Zellhuber N, Helbig R, James P, Bloching M. Cochlear reimplantation from mid-scala to lateral wall electrode array: Surgical and hearing outcome. Clin Case Rep. 2021 Jun 17;9(6): e04210. doi: 10.1002/ccr3.4210. PMID: 34457271; PMCID: PMC8374987.
2. Ketterer MC, Aschendorff A, Arndt S, Beck R. Electrode array design determines scalar position, dislocation rate and angle and postoperative speech perception. Eur Arch Otorhinolaryngol. 2022 Sep;279(9):4257-4267. doi: 10.1007/s00405-021-07160-2. Epub 2021 Nov 15. Erratum in: Eur Arch Otorhinolaryngol. 2021 Dec 16: PMID: 34778920; PMCID: PMC9363302.
3. Helbig S, Adel Y, Leinung M, Stöver T, Baumann U, Weissgerber T. Hearing Preservation Outcomes After Cochlear Implantation Depending on the Angle of Insertion: Indication for Electric or Electric-Acoustic Stimulation. Otol Neurotol. 2018 Aug;39(7):834-841. doi: 10.1097/MAO.0000000000001862. PMID: 29912820.
4. Büchner A, Illg A, Majdani O, Lenarz T. Investigation of the effect of cochlear implant electrode length on speech comprehension in quiet and noise compared with the results with users of electro-acoustic stimulation, a retrospective analysis. PLoS One. 2017 May 15;12(5): e0174900. doi: 10.1371/journal.pone.0174900. PMID: 28505158; PMCID: PMC5432071.
5. O'Connell BP, Hunter JB, Gifford RH, Rivas A, Haynes DS, Noble JH, Wanna GB. Electrode Location and Audiologic Performance After Cochlear Implantation: A Comparative Study Between Nucleus CI422 and CI512 Electrode Arrays. Otol Neurotol. 2016 Sep;37(8):1032-5. doi: 10.1097/MAO.0000000000001140. PMID: 27525618; PMCID: PMC4988342.
6. Buchman CA, Dillon MT, King ER, Adunka MC, Adunka OF, Pillsbury HC. Influence of cochlear implant insertion depth on performance: a prospective randomized trial. Otol Neurotol. 2014 Dec;35(10):1773-9. doi: 10.1097/MAO.0000000000000541. PMID: 25122601.
7. Esquia Medina GN, Borel S, Nguyen Y, Ambert-Dahan E, Ferrary E, Sterkers O, Grayeli AB. Is electrode-modiolus distance a prognostic factor for hearing performances after cochlear implant surgery? Audiol Neurootol. 2013;18(6):406-13. doi: 10.1159/000354115. Epub 2013 Oct 23. PMID: 24157488.
